# Osimertinib induces paraptosis and TRIP13 confers resistance in glioblastoma cells

**DOI:** 10.1038/s41420-023-01632-6

**Published:** 2023-09-05

**Authors:** Lulu Hu, Ji Shi, Dachuan Shen, Xingyue Zhai, Dapeng Liang, Jing Wang, Chunrui Xie, Zhiyu Xia, Jing Cui, Feng Liu, Sha Du, Songshu Meng, Haozhe Piao

**Affiliations:** 1 Institute of Cancer Stem Cell, Dalian Medical University, 116044 Dalian, China; 2 Department of Laboratory Medicine, Affiliated Qingdao Central Hospital, Qingdao University, 266000 Qingdao, China; 3 Department of Neurosurgery, Cancer Hospital of China Medical University, Liaoning Cancer Hospital & Institute, 110042 Shenyang, China; 4 Department of Oncology, Affliated Zhongshan Hospital of Dalian University, 116001 Dalian, China; 5 Clinical Nutrition Department, The Second Hospital of Dalian Medical University, 116023 Dalian, China

**Keywords:** Cancer therapeutic resistance, Apoptosis

## Abstract

The efficacy of osimertinib, a third-generation epidermal growth factor receptor tyrosine kinase inhibitor, has been evaluated in glioblastoma (GBM) through preclinical and clinical trials. However, the underlying mechanism of osimertinib-induced GBM cell death and the underlying resistance mechanism to osimertinib remains unclear. Here, we demonstrate that Osimertinib induces paraptosis in GBM cells, as evidenced by the formation of cytoplasmic vacuoles, accumulation of ubiquitinated proteins, and upregulation of endoplasmic reticulum (ER) stress markers like CHOP. Additionally, neither apoptosis nor autophagy was involved in the osimertinib-induced cell death. RNAseq analysis revealed ER stress was the most significantly downregulated pathway upon exposure to osimertinib. Consistently, pharmacologically targeting the PERK-eIF2α axis impaired osimertinib-induced paraptosis. Notably, we show that the expression of thyroid receptor-interacting protein 13 (TRIP13), an AAA+ATPase, alleviated osimertinib-triggered paraptosis, thus conferring resistance. Intriguingly, MK-2206, an AKT inhibitor, downregulated TRIP13 levels and synergized with Osimertinib to suppress TRIP13-induced high GBM cell growth in vitro and in vivo. Together, our findings reveal a novel mechanism of action associated with the anti-GBM effects of osimertinib involving ER stress-regulated paraptosis. Furthermore, we identify a TRIP13-driven resistance mechanism against Osimertinib in GBM and offer a combination strategy using MK-2206 to overcome such resistance.

## Introduction

Osimertinib, also known as AZD9291, is a third-generation epidermal growth factor receptor tyrosine kinase inhibitor (EGFR-TKI) approved for the treatment of patients with non-small cell lung cancer (NSCLC) with a T790M mutation in EGFR. Osimertinib has demonstrated remarkable efficacy against various cancers in preclinical and clinical trials [1]. *EGFR* is among the most frequently deranged genes in glioblastoma (GBM), making it an attractive therapeutic strategy. However, the approved first and second generations of EGFR-TKIs, such as erlotinib, have shown no significant benefit in patients with GBM [2], partly due to limited blood-brain barrier penetration. On the other hand, osimertinib has shown significant brain penetration [3] and has demonstrated inhibitory activity against GBM in preclinical and clinical trials [4–6], thus offering great potential for the treatment of EGFR-driven GBM. However, the precise mode of Osimertinib-induced cell death in GBM and the underlying mechanisms are unclear. Furthermore, given the potential of primary and/or acquired resistance to osimertinib in GBM, the mechanism underlying the resistance remains to be investigated.

Paraptosis is a non-apoptotic form of cell death characterized by cytoplasmic vacuole formation resulting from the endoplasmic reticulum (ER) and/or mitochondrial swelling [7–9]. Accumulating evidence shows that cytoplasmic vacuolization and mitochondrial swelling/damage are well-known key features of paraptosis [7, 10, 11]. Other established halmarks of paraptosis include caspase independence with an absence of membrane blebbing and DNA condensation/fragmentation, disruption of ER homeostasis, as well as activation of MAPK signaling [7, 10, 11]. Although the biochemical mediators of paraptosis are not yet completely understood, recent studies have shown that paraptosis is associated with the perturbation of cellular proteostasis through proteasome inhibition and the generation of reactive oxygen species (ROS) [12, 13].

Our study is the first to report that osimertinib induces ER stress-related paraptosis in GBM cells. We recently reported that thyroid receptor-interacting protein 13 (TRIP13), an AAA+ATPase, and EGFR form a feed-forward loop promoting GBM growth [14]. Here, we also show that TRIP13 confers resistance to osimertinib in GBM cells.

## Results

### Osimertinib suppresses GBM cell growth in vitro and in vivo

**Fig. 1:**
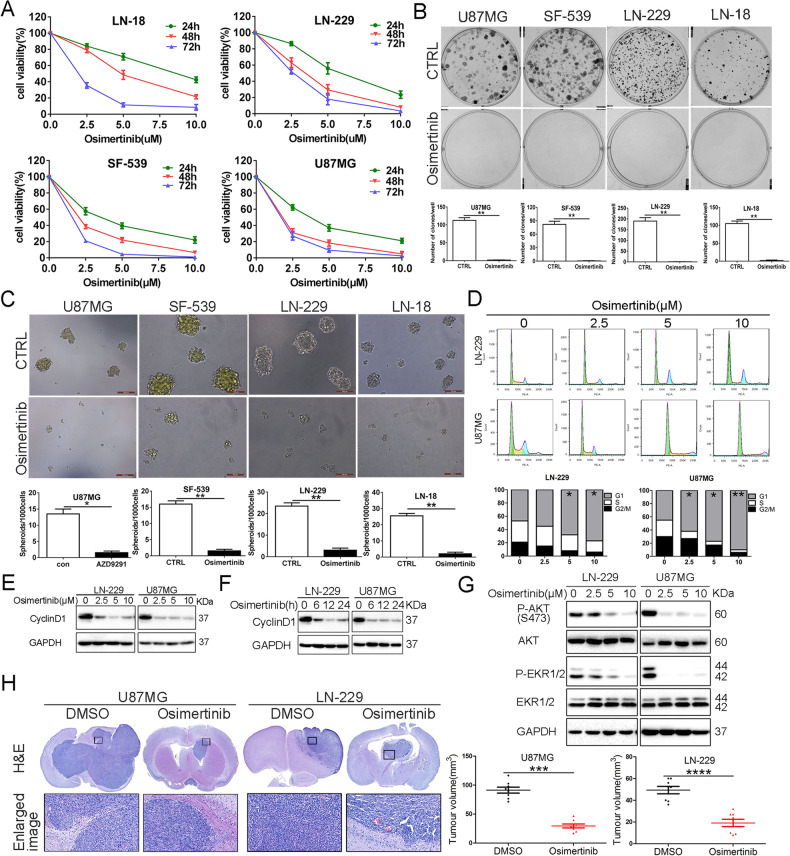
Osimertinib treatment inhibits GBM cell proliferation and induces cell cycle arrest. **A** GBM cell lines (LN-18, LN-229, SF-539, and U87MG) were vehicle-treated or treated with varying concentrations of Osimertinib (2.5, 5, and 10 μM) for 24, 48, 72 h. Cell growth inhibition was determined using a CCK8 assay. **B** GBM Cells (LN-18, LN-229, SF-539, and U87MG) were treated with vehicle or 5 μm Osimertiinb, then cultured in a complete medium for 12 days for colony formation analysis. Results represent as the mean ± SD, *n* = 6, **p < 0.01. **C** GBM cells were pre-treated as in **B** for 24 h and seeded in ultra-low attachment 96-well plates for 7 days. Scale bar = 100 μm. **D** LN-229 and U87MG cells were treated the same as in **A** and were analyzed by FACS after staining with propidium iodide for cell cycle analysis at 24 h. Results represent the mean ± SD, *p < 0.05, **p < 0.01. **E** LN-229 and U87MG cells were treated the same as in **A**, and protein levels of CyclinD1 and GAPDH were analyzed by immunoblot analysis (IB). **F** Time course analysis of levels of CyclinD1 and GAPDH by IB in LN-229 and U87MG cells treated the same as in **B**. **G** LN-229 and U87MG cells were treated the same as in **D**, protein levels of P-AKT(S473), AKT, P-ERK1/2, ERK1/2 and GAPDH were analyzed by IB. **H** Mice’s brain-bearing LN-229 or U87MG xenografts were vehicle-treated or treated with Osimertinib (50 mg/kg) for 2 weeks. Representative images of H&E-stained brain tumor xenografts were shown (**H**). Tumor volume is represented as the mean ± SD, *n* = 7, ****p < 0.0001. Data represent three independent experiments with similar results. *p < 0.05, **p < 0.01, ***p < 0.001, ****p < 0.0001.

We first determined the effect of osimertinib on short-term cell growth in several established GBM cell lines, including LN-18, LN-229, SF-539, and U87MG cells. Osimertinib elicited a half-maximal growth inhibitory concentration (IC_50_) range of 4–7 μM within 24–72 h (Fig. 1A), consistent with a recent report showing that osimertinib suppressed GBM cell growth [4]. Furthermore, osimertinib treatment at a concentration of 5 μM significantly inhibited clonogenic growth and sphere formation of the four GBM cell lines (Fig. 1B, C). Furthermore, osimertinib arrested LN-229 and U87MG cells in the sub G2/M phase in a dose-dependent manner (Fig. 1D). Consistently, exposure to osimertinib induced a dose- and time-dependent reduction in Cyclin D1 levels in both LN-229 and U87MG cells (Fig. 1E, F). As expected, osimertinib exposure significantly reduced the activation of the two main EGFR downstream signaling pathways, i.e., p-AKT and p-ERK1/2, in LN-229 and U87MG cells (Fig. 1G). Furthermore, osimertinib induced a statistically significant reduction in tumor growth in mice brains bearing LN-229 or U87MG xenografts (Fig. 1H), verifying our in vitro findings. Collectively, these data indicate that osimertinib exhibits a potent anti-proliferative efficacy against GBM in vitro and in vivo.

### Osimertinib induces paraptosis-like cell death in GBM cells

**Fig. 2:**
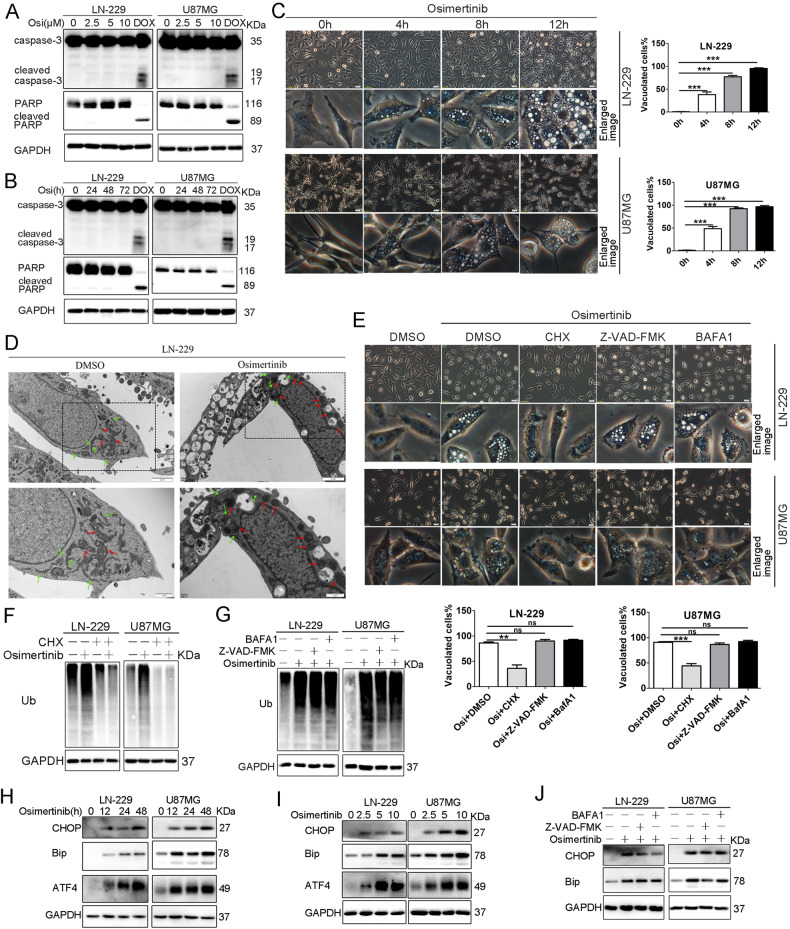
Osimertinib induces vacuolization and paraptosis-like cell death in GBM cells. **A** LN-229 and U87MG cells were treated with vehicle or varying concentrations of Osimertinib for 24 h. Immunoblotting (IB) analysis for Caspase-3, PARP, and GAPDH, 5 μM Dox-treatment as a positive control. **B** LN-229 and U87MG cells were treated with vehicle or 5 μM Osimertinib for varying time. IB analysis for Caspase-3, PARP, and GAPDH, 5 μM Dox-treatment as a positive control. **C** LN-229 and U87MG cells were treated as in **B**. Cell morphology was examined by phase-contrast microscopy in **C**, (scale bar = 20 μm). The numbers of vacuolated and non-vacuolated cells were counted manually, and the ratio of vacuolated cells was calculated and shown as mean ± SD, *n* = 6; ***p < 0.001. **D** Analysis of the morphological changes in Osimertinib-treated cells by electron microscopy Electron microscopy was performed in LN-229 cells treated with 5 μM Osimertinib or DMSO for 24 h. Red arrows indicate dilated mitochondria and green arrows indicate dilated ER. Bar, 1 μm. **E** LN-229 and U87MG cells were exposed to Osimertinib (5 μM) in the absence or presence of CHX (10 μM), Z-VAD-FMK (5 μM), BafA1 (2.5 μM) for 24 h. Cell morphology was examined by phase-contrast microscopy (scale bar = 20 μm). The numbers of vacuolated and non-vacuolated cells were counted manually, and the ratio of vacuolated cells was calculated and shown as mean ± SD, *n* = 6; **p < 0.01, ***p < 0.001. **F** LN-229 and U87MG cells were exposed to Osimertinib (5 μM) in the absence or presence of CHX (10 μM) for 24 h. Cell lysates were analyzed by IB using the indicated antibodies. **G** LN-229 and U87MG cells were exposed to Osimertinib (5 μM) in the absence or presence of Z-VAD-FMK (5 μM), BafA1 (2.5 μM) for 24 h. Cell lysates were analyzed by IB using the indicated antibodies. **H** LN-229 and U87MG cells were treated with vehicle or 5 μM Osimertiinb for the indicated times and cell lysates were analyzed by IB using the indicated antibodies. **I** LN-229 and U87MG cells were vehicle-treated or treated with varying concentrations of Osimertinib (2.5, 5, and 10 μM) for 24 h. Cell lysates were analyzed by immunoblotting (IB) using the indicated antibodies. **J** LN-229 and U87MG cells were treated the same as in **G**, protein levels of CHOP, Bip, and GAPDH were analyzed by IB. All experiments in this figure were performed three times with comparable results.

We next aimed to investigate the mechanism underlying the anti-GBM effects of osimertinib and determine which cell death pathway is involved. Previous studies indicate that osimertinib could trigger apoptosis and/or autophagy in lung and colorectal cancer cell lines [1, 15–17]. We performed immunoblot analysis of LN-229 or U87MG cell lysates treated with osimertinib at various concentrations for 24 h (Fig. 2A) or at 5 μM for up to 72 h (Fig. 2B). We observed no significant change in the levels of caspase-3 processing and poly (ADP-ribose) polymerase (PARP) cleavage, two classical apoptosis markers. As a positive control, doxorubicin (Dox), a known apoptosis inducer, caused marked cleavage of caspase-3 and PARP in these cells (Fig. 2A, B). Similar results were obtained in LN-229 or U87MG cells overexpressing the EGFR variant III (EGFRvIII), a key driver of GBM pathogenesis in over 20% of patients with GBM (Supplementary Fig. 1A).

To determine whether apoptosis is involved in osimertinib-mediated GBM cell death, we used annexin-V staining assay to assess the extent of phosphatidyl-serine externalization. Our analysis revealed no significant change in the percentages of annexin-V-positive cells between mock and osimertinib (5 μM)-treated LN-229 and U87MG cells up to 72 h. By contrast, Dox markedly increased the number of apoptotic cells (Supplementary Fig. 1B). Additionally, osimertinib treatment did not significantly alter the expression levels of LC3II and p62, the canonical autophagy markers, in either LN-229 or U87MG cells (Supplementary Fig. 1C), ruling out autophagy as a potential cause of osimertinib-induced cell death in GBM cells. Consistent with the above results, neither z-VAD-FMK (an apoptosis inhibitor) nor bafilomycin A1 (BafA1, an autophagy inhibitor) significantly affected osimertinib-induced GBM cell death (Supplementary Fig. 1D), further confirming that osimertinib-mediated cell death in GBM cells does not involve apoptosis or autophagy.

To determine the effect of osimertinib on GBM cell morphology, we conducted phase-contrast microscopy on LN-229, U87MG, LN-18, and SF-539 cell lines treated with osimertinib. Our results revealed that osimertinib treatment led to a time-dependent accumulation of cytoplasmic vacuoles in these GBM cell lines (Fig. 2C and Supplementary Fig. 1E). In contrast, cell membrane and nuclei of these osimertinib-treated cells remained largely intact. To investigate the origin(s) of these cytoplasmic vacuoles, we performed electron microscopy analysis, which revealed swollen mitochondria and dilated ER structures in osimertinib-treated LN-229 cells (Fig. 2D).

To examine whether autophagy is involved in osimertinib-induced vacuoles, we silenced ATG7, an essential for autophagy inducer, using lentiviral delivery of short hairpin RNAs (shRNAs) in LN-229 and U87MG cells and examined the formation of vacuoles. Osimertinib triggered the formation of vacuoles in ATG7-depleted cells as in control cells, suggesting that osimertinib-induced vacuoles in these cells might be independent of autophagy (Supplementary Fig. 1F).

Studies show that cytoplasmic vacuolization in the absence of caspase activation and apoptotic marker protein expression is characteristic of paraptosis [12, 18, 19], and the induction of paraptosis is known to require protein synthesis [7, 20, 21]. Indeed, pretreatment of the tested GBM cell lines with the protein synthesis blocker cycloheximide (CHX) effectively decreased osimertinib-induced formation of massive vacuoles, while pretreatment with either Z-VAD-FMK or BafA1 had no effect (Fig. 2E and Supplementary Fig. 1G), indicating that protein synthesis is required for osimertinib-triggered vacuolization [22]. Consistently, CHX treatment significantly attenuated osimertinib-triggered GBM cell death (Supplementary Fig. 1H).

Studies show that cytoplasmic vacuolation predominantly derived from ER stress, lacked caspase activation, and increased protein ubiquitination [12, 13, 18, 23]. Indeed, a 24 h incubation of osimertinib in LN-229 and U87MG cells greatly enhanced the number of ubiquitinated proteins (Fig. 2F), an effect alleviated by the pretreatment with CHX but not BafA1 or Z-VAD-FMK (Fig. 2G). The accumulation of misfolded and unfolded proteins may result in increased ER stress [24]. Immunoblotting analysis revealed that osimertinib treatment upregulated the protein levels of several key players and biomarkers of ER stress, including CHOP, GRP78/Bip, and ATF4, in a time- and dose-dependent manner in both LN-229 and U87MG cells (Fig. 2H, I). Furthermore, Osimertinib treatment increased the protein levels of CHOP in spheroids derived from LN-229 or U87MG cells (Supplementary Fig. 1I). Notably, neither BafA1nor Z-VAD-FMK could markedly prevent osimertinib-induced upregulation of CHOP and Bip in LN-229 and U87MG cells (Fig. 2J). Collectively, our findings suggest that osimertinib-induced cell death in the GBM cells shares both the morphological and biochemical features of paraptosis.

### Deregulated ER stress contributes to osimertinib-induced paraptosis

**Fig. 3:**
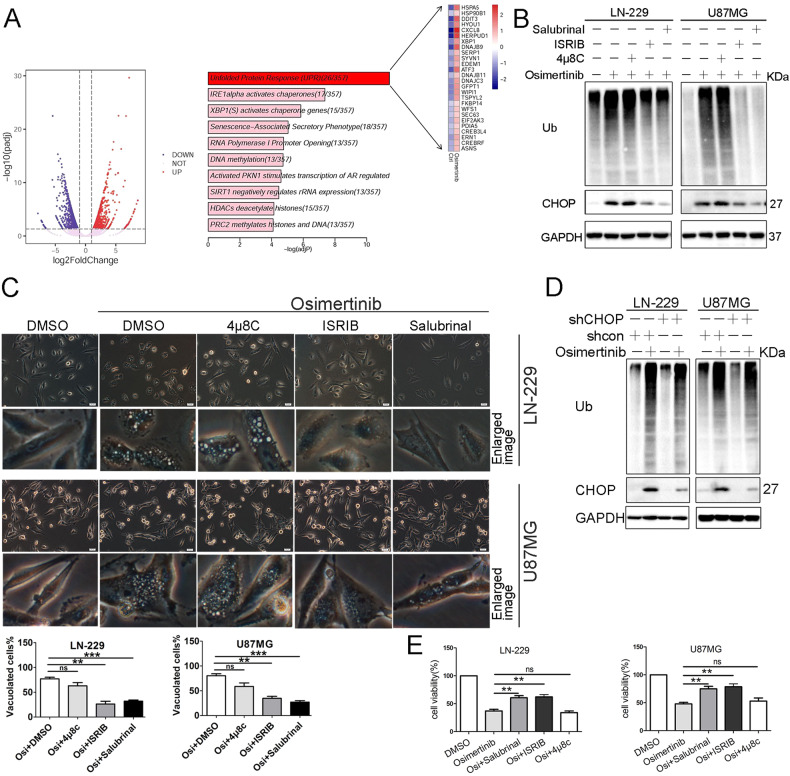
ER stress is involved in Osimertinib-induced paraptosis. **A** Volcano plot of differentially expressed genes (DEGs) in Osimertinib-treated LN-229 and vehicle-treated control cells (left panel). The upregulated genes (UP) were depicted by red points (corrected *p*-value < 0.05, log2Fold-Change > 1). The downregulated genes (DOWN) were depicted by blue points (corrected *p*-value < 0.05, log2Fold-Change < −1). The pink points stand for genes that were not significantly differentially expressed (NOT). Reactome pathway enrichment analysis for upregulated genes (right panel). Top 10 enriched pathways were shown. The *x*-axis represents the corrected *p*-value, the number of genes mapping to the corresponding enriched pathways was labeled in parentheses. Representative genes belonging to the highest-ranking pathway (unfolded protein response (UPR)) were highlighted. The heatmap shows the per-row mean centered gene expression log2 (FPKM + 1) values for UPR genes. **B**, **C** LN-229 and U87MG cells were exposed to Osimertinib (5 μM) in the absence or presence of Salubrinal (5 μM), 4 μ8C (2.5 μM), ISRIB (2.5 μM) for 24 h. Cell lysates were analyzed by immunoblotting (IB) using the indicated antibodies (**B**) and cell morphology was examined by phase-contrast microscopy (**C**) (scale bar = 20 μm). The numbers of vacuolated and non-vacuolated cells were counted manually, and the ratio of vacuolated cells was calculated and shown as mean ± SD, *n* = 6; **p < 0.01, ***p < 0.001. **D** GBM cells (LN-229-shcon, LN-229-shCHOP, U87MG-shcon, U87MG-shCHOP) were vehicle-treated or treated with Osimertinib (5 μM) for 24 h. Cell lysates were analyzed by IB using the indicated antibodies. **E** LN-229 and U87MG cells were treated the same as in **B**, and cell growth was determined by CCK-8 cell survival assay. The results were shown as mean ± SD, *n* = 6; **p < 0.01. Data represent three independent experiments with similar results.

To gain insight into the mechanisms underlying osimertinib-induced paraptosis, we performed RNA sequencing (RNAseq) analysis to compare gene expression profiles between osimertinib-treated LN-229 cells and cells treated with vehicle. Raw data have been deposited in the Genome Sequence Archive (https://ngdc.cncb.ac.cn/gsa/) under submission number PRJCA006774. Volcano plot revealed 1858 differentially expressed genes (DEGs) between osimertinib-treated LN-229 and vehicle-treated control cells, with 867 upregulated and 991 downregulated DEGs in Osimertinib-treated LN-229 cells (Fig. 3A). Reactome pathway analysis of the upregulated DEGs revealed significant enrichment of pathways related to the unfolded protein response (UPR), IRE1 alpha activated chaperones, and XBP1(S) activated chaperone genes (Fig. 3A), suggesting close links with protein processing in the endoplasmic reticulum (ER) and ER stress processes. To investigate the role of ER stress in osimertinib-induced paraptosis, we examined the activity of PRKR-like endoplasmic reticulum kinase (PERK)-eIF2α axis, or inositol-requiring enzyme 1 alpha (IRE1α), two major upstream players in ER stress pathways. Pretreatment with compounds targeting PERK (ISRIB) or eIF2α (Salubrinal) substantially attenuated osimertinib-induced CHOP expression (Fig. 3B), accumulation of polyubiquitinated proteins (Fig. 3B), and vacuolization (Fig. 3C) in both LN-229 and U87MG cells. However, pretreatment with the IRE1α inhibitor 4μ8C failed to do so.

Additionally, shRNA-mediated knockdown of CHOP in LN-229 and U87MG cells did not alter the osimertinib-induced increase in the level of polyubiquitinated proteins (Fig. 3D), indicating that osimertinib blocked proteasomal activity upstream of CHOP expression. Furthermore, both ISRIB and Salubrinal significantly alleviated the amount of cell death caused by osimertinib in LN-229 and U87MG cells (Fig. 3E), whereas 4 μ8C had no impact. Collectively, our data indicate that the PERK- eIF2α arm of the ER stress pathway plays a critical role in osimertinib-induced paraptosis.

### TRIP13 confers resistance to osimertinib-induced paraptosis

**Fig. 4:**
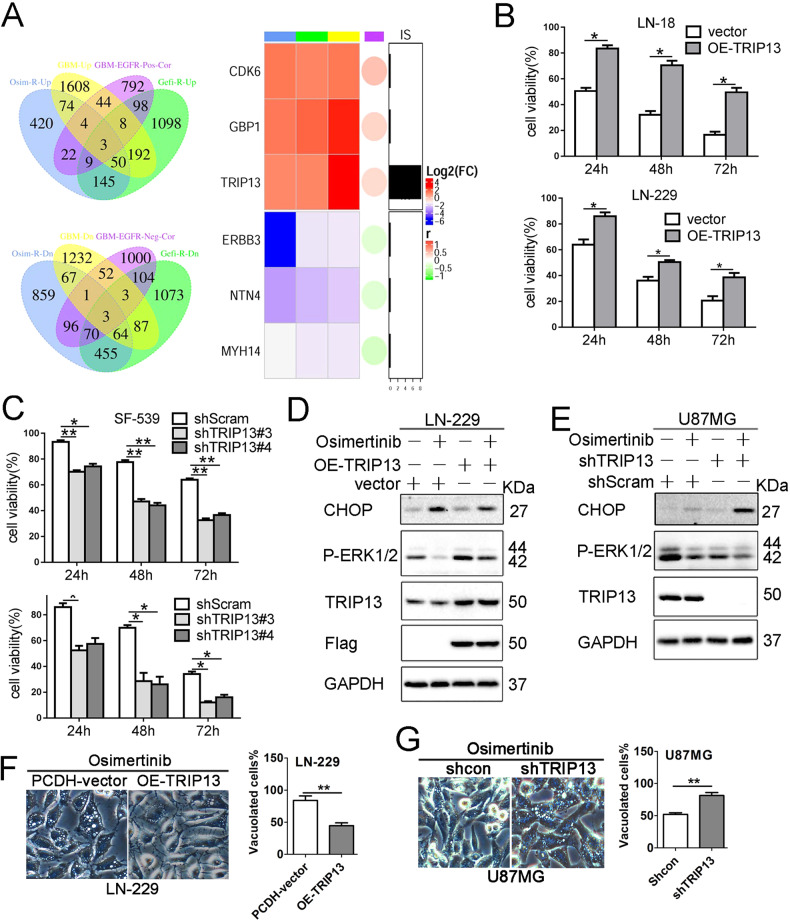
TRIP13 expression regulates GBM cell sensitivity to Osimertinib. **A** Integrating multiple layers of genomic data to identify TRIP13 as a potential regulator of intrinsic EGFR-TKI resistance in GBM. Venn diagrams showing the overlap of Osimertinib-resistance (Osim-R-Up/Dn) genes, Gefitinib-resistance (Gefi-R-Up/Dn) genes, GBM-dysregulated genes (GBM-Up/Dn), and EGFR co-expressed genes (GBM-EGFR-Pos/Neg-Cor). Heatmap visualization of six common overlapping genes. The color bar denotes the Log2 (Fold-Change (FC)) of differentially expressed genes in GSE172002, TCGA-GBMLGG rnaseq datasets and pearson correlation (*r*) of EGFR co-expressed genes in CPTAC-GBM TMT-based proteomics dataset. Barplot represents log10 (Intensity Score (IS)) of EGFR-binding partner identified in our previously reported CoIP-MS dataset. **B** LN-229 and LN-18 cells with or without TRIP13 overexpression were treated with Osimertinib (5 μM) for the indicated times, and cell growth was determined by CCK-8 cell survival assay. The results were shown as mean ± SD, *n* = 6; *p < 0.05. **C** SF-539 and U87MG cells with or without TRIP13 knockdown were treated with Osimertinib (5 μM) for the indicated times, and cell growth was determined by CCK-8 cell survival assay. The results was shown as mean ± SD, *n* = 6; *p < 0.05, **p < 0.01. **D** LN-229 cells with or without TRIP13 overexpression were vehicle-treated or treated with Osimertinib (5 μM) for 24 h. Cell lysates were analyzed by immunoblotting (IB) using the indicated antibodies. **E** U87MG cells with or without TRIP13 knockdown were vehicle-treated or treated with Osimertinib (5 μM) for 24 h. Cell lysates were analyzed by IB using the indicated antibodies. **F** LN-229 cells with or without TRIP13 overexpression were treated the same as in **D**, and cell morphology was examined by phase-contrast microscopy (scale bar = 20 μm). The numbers of vacuolated and non-vacuolated cells were counted manually, and the ratio of vacuolated cells was calculated and shown as mean ± SD, *n* = 6, **p < 0.01. **G** U87MG cells with or without TRIP13 knockdown were treated the same as in **E**, and cell morphology was examined by phase-contrast microscopy (scale bar = 20 μm). The numbers of vacuolated and non-vacuolated cells were counted manually, and the ratio of vacuolated cells was calculated and shown as mean ± SD. *n* = 6, **p < 0.01.Data represent three independent experiments with similar results.

To explore potential regulators of resistance to EGFR-TKIs in GBM, we integrated multiple layers of genomic datasets and identified six potential regulators (CDK6, GBP1, TRIP13, ERBB3, NTN4, MYH14) of intrinsic EGFR-TKIs resistance in GBM (Fig. 4A). All the candidate genes fulfilled the following criteria: (a) Differentially expressed between osimertinib-resistant and osimertinib-sensitive cells in a GEO RNAseq dataset (GSE172002). (b) Differentially expressed between gefitinib-resistant and gefitinib-sensitive cells in a GEO RNAseq dataset (GSE172002). (c) Aberrantly expressed in GBM, compared with LGG, in TCGA-GBM/LGG RNAseq datasets. (d) Highly co-expressed with EGFR in CPTAC-GBM TMT-based proteomics dataset.

To further investigate the role of these potential regulators in EGFR-TKI resistance, we reanalyzed our in-house EGFR Co-IP-MS dataset and found that TRIP13 was the only EGFR-binding partner among all the six potential regulators. Furthermore, we recently reported that TRIP13 potentiates EGFR signaling to promote GBM progression [14]. We thus hypothesized that TRIP13 might be employed as a resistance mechanism to Osimertinib in GBM.

To test our hypothesis, we ectopically expressed Flag-tagged TRIP13 in LN-18 and LN-229 cells and found that these cells exhibit significant resistance to osimertinib compared to control cells, as determined by a CCK-8 assay (Fig. 4B). Conversely, shRNA-mediated depletion of TRIP13 in U87MG and SF-539 cells rendered these cells more sensitive to osimertinib, compared to control cells (Fig. 4C).

We next examined whether TRIP13 expression affects osimertinib-induced CHOP expression. We found an increase in the CHOP level in U87MG cells upon TRIP13 depletion, whereas it decreases in osimertinib-treated LN-229 cells with TRIP13 overexpression (Fig. 4D, E). As expected, the downregulated phosphorylation levels of ERK1/2 induced by osimertinib in GBM cells were robustly reversed by TRIP13 overexpression in LN-229 cells (Fig. 4D), whereas enhanced by TRIP13 depletion in U87MG cells (Fig. 4E). Furthermore, TRIP13 overexpression in LN-229 cells alleviated (Fig. 4F) the formation of cytoplasmic vacuoles induced by osimertinib, while they were enhanced by TRIP13 depletion in U87MG cells (Fig. 4G). Together, these findings suggest that TRIP13 might interfere with osimertinib-induced paraptosis in GBM cells, conferring resistance to osimertinib.

### AKT inhibitor MK-2206 effectively overcomes TRIP13-mediated intrinsic resistance of GBM cells to osimertinib

**Fig. 5:**
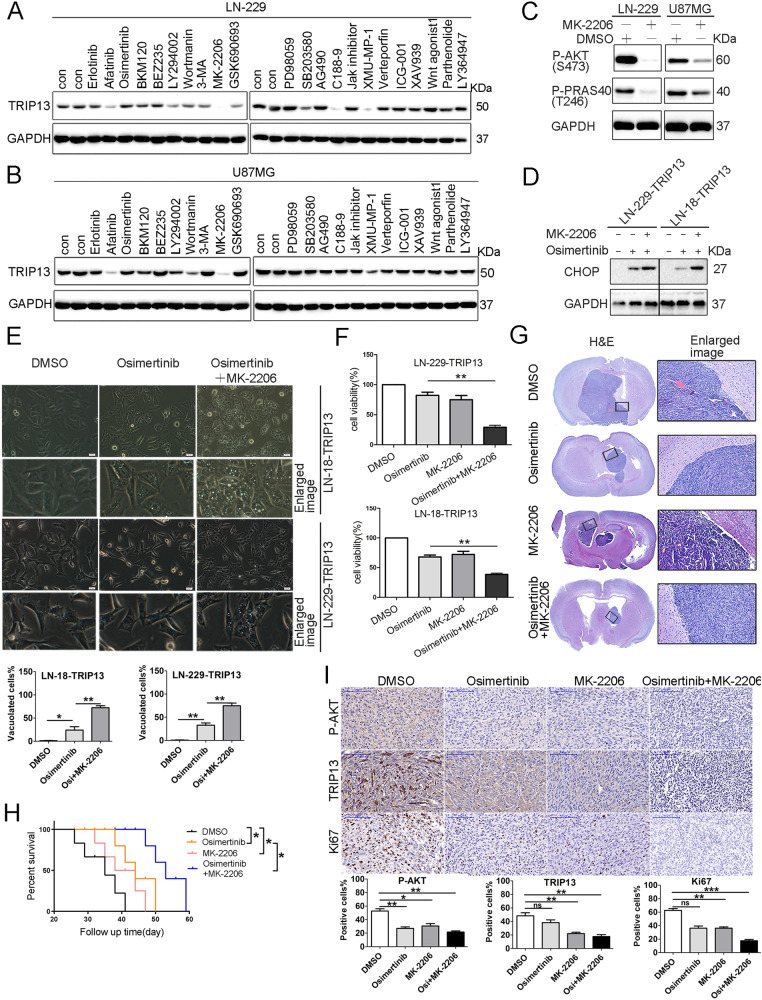
MK-2206 suppresses TRIP13-overexpressing GBM cell growth. **A**, **B** LN-229 or U87MG cells were exposed to EGFR inhibitor Afatinib (5 μM), Erlotinib (10 μM), and Osimertinib (5 μM), PI3K/AKT inhibitors BKM120(2.5 μM), BEZ235(2.5 μM), LY294002(10 μM), Wortmanin(5 μM), 3-Methyladenine (3-MA)(10 μM), MK-2206(5 μM), and GSK690693(10 μM), MEK/ERK inhibitor PD98059(10 μM), p38MAPK inhibitor SB203580(10 μM), JAK/STAT3 inhibitor C188-9(5 μM), Hippo inhibitors XMU-MP-1 (5 μM) and Vertepofin inhibitor (10 μM), Wnt inhibitor ICG-001(10 μM), XAV939(5 μM) and Wnt agonist 1(5 μM), NF-kB inhibitor Parthenolide(2.5 μM), and TGFβ inhibitor LY364947(10 μM). Cell lysates were analyzed by immunoblotting (IB) using the indicated antibodies. **C** LN-229 and U87MG cells were treated with vehicle or 5μm MK-2206 for 24 h and cell lysates were analyzed by IB using the indicated antibodies. **D**–**F** LN-229 and LN-18 cells overexpressing TRIP13 were exposed to Osimertinib (5 μM) in the absence or presence of MK-2206 (5 μM) for 24 h. Cell lysates were analyzed by IB using the indicated antibodies (**D**), cell morphology was examined by the phase-contrast microscopy (scale bar = 20 μm). The numbers of vacuolated and non-vacuolated cells were counted manually, and the ratio of vacuolated cells was calculated and shown as mean ± SD, *n* = 6; *p < 0.05, **p < 0.01 (**E**). The cell growth was determined by CCK-8 cell survival assay (**F**) (**p < 0.01). **G**–**I** Combined treatment of Osimertinib and MK-2206 suppressed tumor growth in an orthotopic GBM model. Mice brain-bearing xerografts derived from LN-229 cells overexpressing TRIP13 were treated with Osimertinib (50 mg/kg) in the absence or presence of MK-2206 (50 mg/kg). Representative images of H&E-stained brain tumor xenograft were shown (**G**). Combined treatment of Osimertinib and MK-2206 prolonged survival. The median survival periods of mice in the control group, Osimertinib group, MK-2206 group, and combination therapy group were 35 days, 44 days, 38 days, and 46 days, respectively. (**H**). IHC staining of the mouse tumor tissues was performed with the indicated antibodies. Representative images are shown (**I**). Scale bar = 100 μm. The numbers of positive cells were counted manually, and the ratio of positive cells was calculated and shown as mean ± SD, *n* = 7; *p < 0.05, **p < 0.01. Data represent three independent experiments with similar results.

Based on the above findings, we hypothesized that TRIP13 downregulation in GBM cells might overcome TRIP13-mediated intrinsic resistance to osimertinib. Therefore, we sought to screen pathway inhibitors that can suppress TRIP13 expression in GBM cells. The tested inhibitors and activators targeted EGFR (Afatinib and Erlotinib), PI3K/AKT (BKM120, BEZ235, LY294002, Wortmanin, 3-MA, MK-2206, and GSK690693), MEK/ERK (PD98059), p38MAPK (SB203580), JAK/STAT3 (C188-9), Hippo (XMU-MP-1and Verteporfin), Wnt (ICG-001 and XAV939), Wnt agonist1, NF-κB (Parthenolide) and TGFβ (LY364947). The specificity of the compounds and appropriate concentrations were confirmed by immunoblotting analyses for the respective phospho-proteins or targeted proteins (data not shown). Among the tested inhibitors, only MK-2206 significantly decreased TRIP13 levels in both LN-229 and U87MG cells (Fig. 5A, B). Consequently, MK-2206 robustly decreased the basal phosphorylation levels of AKT (S473) and its substrate PRAS40 (T246) in these cells (Fig. 5C). Furthermore, MK-2206 treatment antagonized TRIP13 overexpression-induced CHOP downregulation and vacuolization in osimertinib-treated LN-18 and LN-229 cells (Fig. 5D, E). Notably, MK-2206 synergized with osimertinib to suppress the growth of TRIP13-overexpressing LN-18 and LN-229 cells (Fig. 5F).

We next examined the efficacy of MK-2206 in combination with osimertinib in an orthotopic GBM xenograft model. LN-229 cells overexpressing TRIP13 were intracranially injected into the brain of immunodeficient mice. Co-treatment of MK-2206 and osimertinib suppressed tumor growth more efficiently and significantly prolonged the survival than either drug alone without notable toxicity (Fig. 5G, H). IHC analysis revealed decreased Ki67 and TRIP13 staining in tumor sections from mice treated with a combination of MK-2206 and osimertinib, compared with drug alone group (Fig. 5I).

## Discussion

The current study shows that osimertinib induces paraptosis, a non-apoptotic cell death, in GBM cells. We have further demonstrated the role of ER stress in osimertinib-induced paraptosis and revealed a previously unrecognized TRIP13-mediated resistance to osimertinib in GBM. To our knowledge, this is the first report uncovering the mechanism underlying the antitumor efficacy of osimertinib in GBM.

Osimertinib, an EGFR-TKI, is a highly effective drug that can penetrate the brain and has shown promising results against GBM in preclinical and clinical studies [4–6, 25]. Encouragingly, a clinical trial with osimertinib on GBM is currently ongoing (NCATS 1-UH2-TR001370-01). However, the mechanisms underlying Osimertinib-mediated growth inhibition in GBM are less understood. We found that Osimertinib did not induce apoptosis or autophagy in GBM cells, unlike what has been observed in colon and lung cancers [1, 15]. Instead, osimertinib induced massive cytoplasmic vacuoles accompanied by the accumulation of ubiquitinated proteins as well as the upregulation of ER stress markers like CHOP in GBM cells. Interestingly, these effects were alleviated by CHX but not by inhibitors of apoptosis and autophagy. In the absence of caspase activation and apoptotic marker expression, cytoplasmic vacuolization is a key characteristic of paraptosis [19, 26]. Therefore, our findings support that osimertinib induces paraptosis in GBM cells. Intriguingly, a previous study found that osimertinib increased the formation of cytoplasmic vacuoles in various cancer cell lines [27]; however, whether the osimertinib-induced vacuolization in these cancer cells is associated with cell death has not been investigated sufficiently. The formation of cytoplasmic vacuoles and the accumulation of ubiquitinated proteins during paraptosis are tightly associated with ER stress. Our RNAseq analysis confirmed the activation of the ER stress along with the unfolded protein response in osimertinib-treated GBM cells. Specifically, we showed that the PERK-eIF2α axis, but not the IRE1α signaling, contributed to osimertinib-induced paraptosis. However, the precise role of the PERK-eIF2α axis in this process needs further examination, as pharmacological compounds that either activate or inhibit eIF2α exhibited similar effects. Therefore, genetic targeting of the PERK-eIF2α axis might provide more insights into its function in osimertinib-induced paraptosis. Furthermore, a recent study reported an increase in IRE1α expression in osimertinib-resistant lung cancer cells, whose growth was suppressed by targeting IRE1α [28].

So far, our understanding of the recognized resistance mechanisms against osimertinib, such as secondary resistance mutations like EGFR C797S [29], or the activation of alternative signaling pathways [30], is largely from studies in lung cancer [31–33]. However, the mechanisms underlying osimertinib resistance in GBM have not yet been extensively explored [5]. However, recent research has suggested that TRIP13 expression may play a role in promoting GBM growth [14, 34]. TRIP13 has also been implicated in a variety of cancers [35, 36]. In line with our previous findings that TRIP13 stabilizes EGFR and potentiates EGFR signaling in GBM [14], our current data demonstrated that TRIP13 expression alleviates osimertinib-induced paraptosis in GBM cells, thereby promoting resistance against osimertinib. Therefore, we first characterized TRIP13 as a therapeutic resistance mechanism against Osimertinib in GBM and further suggested high TRIP13 expression as a predictive marker for osimetinib resistance. However, this hypothesis requires further examination in clinical trials.

Our findings also suggest that TRIP13 might be a potential therapeutic target for overcoming osimertinib resistance in GBM. We found that MK-2206, an AKT inhibitor substantially decreased TRIP13 expression in GBM cells. Notably, the combination of osimertinib and MK-2206 shows synergistic effects in suppressing TRIP13-high GBM cell growth in vitro and in vivo. Clinical trials with MK-2206 have been completed in several types of cancer, including advanced breast cancer (NCT01277757) and metastatic neuroendocrine tumors (NCT01169649). In addition, clinical trials with the combination of MK-2206 and EGFR TKIs, such as erlotinib (OSI-774) and gefitinib, have been completed in NSCLC (NCT01294306 and NCT01147211), suggesting a potential combination strategy that might be utilized for the treatment of GBM. While previous investigations have failed to extend glioma patient survival using either EGFR-targeting agents or inhibitors of PI3K pathway components [37], we believe our findings are meaningful in designing clinical trials with osimertinib and MK-2206 for osimertinib-resistant GBM patients.

Collectively, this study provides the first evidence that osimertinib induces ER stress-related paraptosis in GBM cells, reveals a TRIP13-driven resistance mechanism against osimeritinib, suggesting that the AKT inhibitor MK-2206 might overcome this resistance.

## Materials and methods

### Cell lines, reagents, and antibodies

Established human GBM cell lines LN-18, LN-229, U87MG (American Type Culture Collection), SF-539 (cell bank of the Chinese Academy of Science), and TRIP13-overexpressing or knockdown cell lines [14], were routinely maintained in minimum essential medium or Dulbecco’s modified Eagle medium with 10% fetal bovine serum (FBS). All cells have passed the mycoplasma contamination test. All reagents and antibodies used in this study are listed in Supplementary Table 1.

### Cell viability, colony formation, and sphere formation assays

Cell viability was assessed using a CCK8 kit (MCE/Y-K0301) according to the manufacturer’s protocol. Colony formation and 3D culture assays were performed as previously described [38, 39].

### Flow cytometric analysis of cell cycle and apoptosis

Cell cycle and apoptosis were analyzed using flow cytometry as previously documented [38].

### Transmission electron microscopy

The morphology of glioma cells was determined at 80 kV with a JEOL 1200EX transmission electron microscope. Three fields containing more than 5 randomly selected microscopy-captured images were examined.

### Immunoblotting assay

Cells were treated with various agents, collected, and processed for immunoblotting analysis as previously described [14]. The densitometries of protein bands were determined with a calibrated GS-670 densitometer to quantify changes.

### Bioinformatics analysis

FPKM expression data in osimertinib-resistant and osimertinib-sensitive cells were obtained from the gene expression omnibus (GEO) data repository with accession number GSE172002. Genes with more than a 1.7-fold-change in FPKM expression level were defined as differentially expressed genes (DEGs). To explore gene expression patterns in gliomas, TCGA-GBMLGG RNAseq and corresponding clinical data were obtained from UCSC XENA (https://xenabrowser.net/datapages/). The DEGs between GBM and LGG were determined using the non-parametric Mann-Whitney test. Genes with absolute fold-change > 2 and *p*-value < 0.05 were considered DEGs. Relative protein abundance data (Unshared log TMT ratio) for patients with GBM were obtained from the Clinical Proteomic Tumor Analysis Consortium (CPTAC, https://cptac-data-portal.georgetown.edu/). Data were normalized using the median centering method to correct for sample loading differences. The *k*-nearest neighbor (k-NN) imputation method was applied to impute the missing values using the impute package in R. Spearman correlation between the protein expression level of EGFR, and its co-expressed genes were calculated, considering genes with an absolute correlation coefficient >0.2 and a *p*-value < 0.05 as EGFR co-expressed genes. Ensemble IDs and gene symbols were converted to Entrez Gene IDs as central identifiers to facilitate joint analysis and cross-dataset comparisons.

### Mouse xenograft experiments and immunohistochemistry

All animal studies were conducted according to the ethical guidelines approved by the Animal Ethics Committee of Dalian Medical University and were performed in accordance with the rules of the SPF Experimental Animal Center of Dalian Medical University. A GBM orthotopic mouse model was established as previously described [14]. The experimental nude mice were randomly divided into groups and each group consisted of 7 nude mice. Briefly, 1 × 10^5^ GBM cells were stereotactically implanted into the frontal lobe of the 5-week-old male athymic Balb/c nude mice (depth 5 mm). Tumor-bearing mice were sacrificed with ether anesthesia, and bioluminescence imaging was performed. Immunohistochemistry was performed as described in our previous work [14]. The processed sections were blocked with 3% BSA and incubated with antibodies against TRIP13, p-AKT, or Ki67. All testing and data analysis were conducted in a blinded manner.

### Statistical analysis

All the experiments were performed at least three times, independently, and are expressed as “mean±s.d.”. We removed a maximum value and a minimum value when calculating the results and the criteria Were pre-established. All testing and data analysis were conducted in a blinded manner. Treatments and control groups were assessed and analyzed with a one-way analysis of variance (ANOVA). Multiple comparisons between the treatment groups and controls were performed using Dunnett’s least significant difference (LSD) test. Statistical significance between groups was calculated using the LSD test in SPSS 17.0 software (SPSS Inc., Chicago, IL, USA). *p* ≤ 0.05 was considered statistically significant.

### Supplementary information

Supplementary Figure

Supplementary Table 1

Original Data File

## Data Availability

The data used and analyzed in this study are available from the corresponding authors upon reasonable request.
