# Supplementary material for: Osimertinib induces paraptosis and TRIP13 confers resistance in glioblastoma cells
Source: Cell Death Discov. 2023 Sep 5;9:333. doi: 10.1038/s41420-023-01632-6 (PMC10480197; doi:10.1038/s41420-023-01632-6)
Supplement: Supplementary file 1 — Supplementary Figure [file 41420_2023_1632_MOESM1_ESM.docx]

**Supplementary Figures 1**


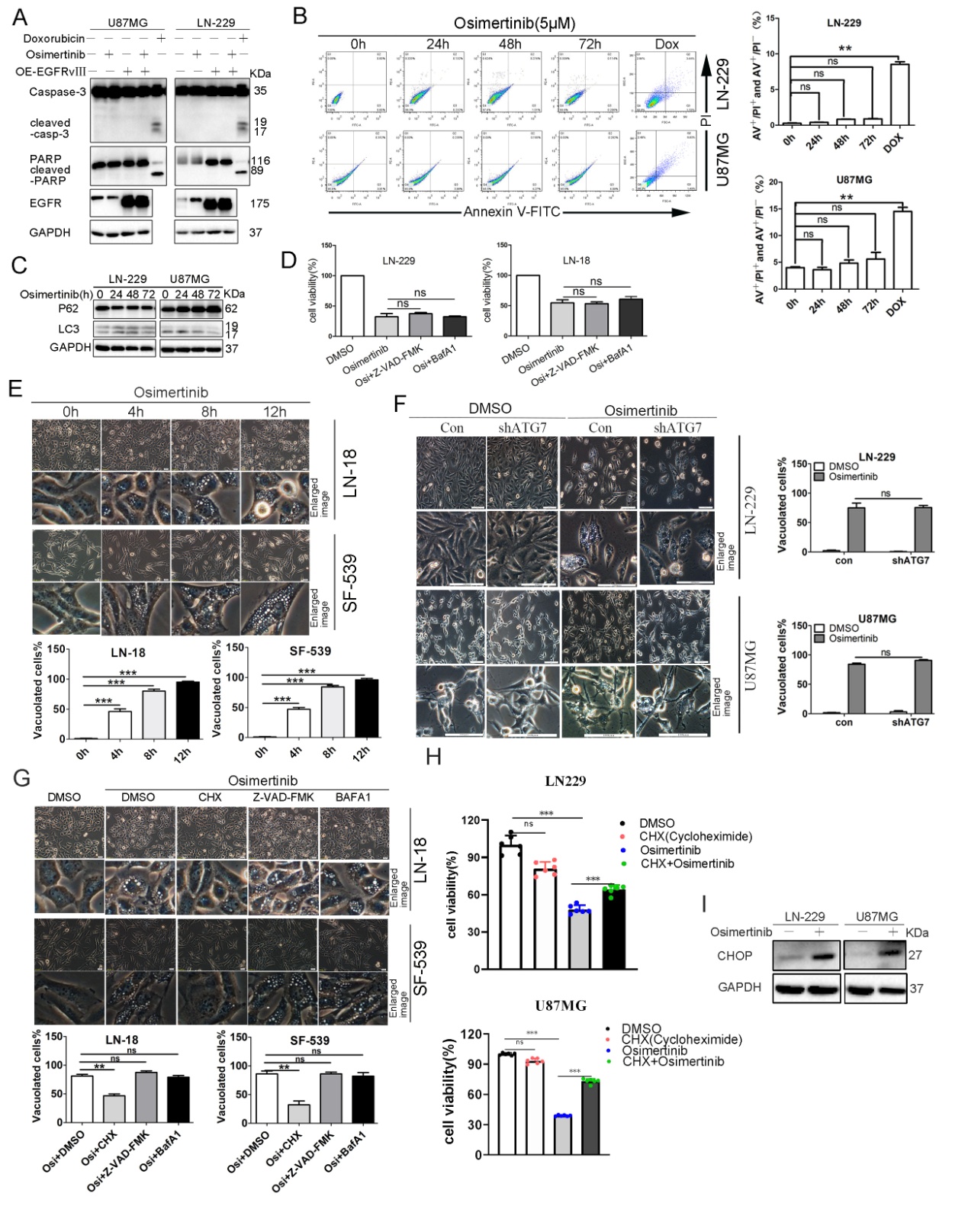


**Supplementary Fig.1** Osimertinib triggers caspase-independent cell death and ER vacuolization in GBM cells. (**A**) LN-229 and U87MG cells with or without EGFRvIII overexpression were treated with Osimertinib (5 μM) or Doxorubicin (5 μM) for 24h, and cell lysates were analyzed by immunoblotting (IB) using the indicated antibodies. (**B**) LN-229 and U87MG cells were treated with vehicle or 5 μM Osimertinib for varying time. Cells were stained using Annexin V/PI double-staining and analyzed by FACS, 5 μM Doxorubicin (Dox)-treatment as a positive control. (**C**) LN-229 and U87MG cells were treated with vehicle or 5μm Osimertinib for the indicated times and cell lysates were analyzed by IB using the indicated antibodies. (**D**) LN-229 and U87MG cells were exposed to Osimertinib (5 μM) in the absence or presence of Z-VAD-FMK (5μM), BafA1 (2.5μM) for 24h. Cell growth was determined by CCK-8 cell survival assay. (**E**) LN-18 and SF-539 cells were treated with vehicle or 5μM Osimertinib for varying time. Cell morphology was examined by phase-contrast microscopy (scale bar=20 μm). The numbers of vacuolated and non-vacuolated cells were counted manually, and the ratio of vacuolated cells was calculated and shown as mean±SD, n =6; ***p < 0.001. (**F**) LN-229 and U87MG cells with or without TRIP13 knockdown were vehicle-treated or treated with Osimertinib (5μM) for 12h. Cell morphology was examined by phase-contrast microscopy (scale bar=20 μm). The numbers of vacuolated and non-vacuolated cells were counted manually, and the ratio of vacuolated cells was calculated and shown as mean±SD, n =6. (**G**) LN-18 and SF-539 cells were exposed to Osimertinib (5 μM) in the absence or presence of CHX (10 μM), Z-VAD-FMK (5μM), BafA1 (2.5μM) for 24h. Cell morphology were examined by phase-contrast microscopy (scale bar=20 μM). The numbers of vacuolated and non-vacuolated cells were counted manually, and the ratio of vacuolated cells was calculated and shown as mean±SD, n =6; **p＜0.01. (**H**) LN-229 and U87MG cells were exposed to Osimertinib (5 μM) in the absence or presence of CHX (10 μM) for 24h. Cell growth was determined by CCK-8 cell survival assay. ***p＜0.001. (**I**) LN-229 and U87MG spheroids were exposed to Osimertinib (5 μM) for 72h. Cell lysates were analyzed by IB using the indicated antibodies. Data represent three independent experiments with similar results.
