# Supplementary material for: Osimertinib induces paraptosis and TRIP13 confers resistance in glioblastoma cells
Source: Cell Death Discov. 2023 Sep 5;9:333. doi: 10.1038/s41420-023-01632-6 (PMC10480197; doi:10.1038/s41420-023-01632-6)
Supplement: Supplementary file 2 — Supplementary Table 1 [file 41420_2023_1632_MOESM2_ESM.docx]

**Supplementary Table 1:** The Reagents used in this project.

| The Reagents used in this project. | | |
| --- | --- | --- |
| REAGENT | SOURCE | Catalog |
| B27 | Gibco | Cat#17504044 |
| bFGF | Millipore | Cat#GF003 |
| EGF | Gibco | Cat#PHG0311 |
| Erlotinib | Selleck | Cat#S1023 |
| Afatinib | Selleck | Cat#S1011 |
| Osimertinib | Selleck | Cat#S7297 |
| BKM120 | Selleck | Cat#S2247 |
| BEZ235 | Selleck | Cat#S1009 |
| LY294002 | Selleck | Cat#S1105 |
| Wortmanin | Calbiochem | Cat#681675 |
| 3-Methyladenine | Sigma | Cat#M9281 |
| MK-2206 | Selleck | Cat#S1078 |
| GSK690693 | Selleck | Cat#S1113 |
| PD98059 | Selleck | Cat#S1177 |
| SB203580 | CALBIOCHEM | Cat#559389 |
| AG490 | CALBIOCHEM | Cat#658401 |
| C188-9 | Selleck | Cat#S8605 |
| JAK inhibitor | CALBIOCHEM | Cat#420099 |
| XMU-MP-1 | Selleck | Cat#S8334 |
| Verteporfin | Selleck | Cat#S1786 |
| ICG-001 | Selleck | Cat#S2662 |
| XAV939 | Selleck | Cat#S1180 |
| Wnt agonist 1 | Selleck | Cat#S8178 |
| Parthenolide | Selleck | Cat#S2341 |
| LY364947 | Selleck | Cat#S2805 |
| MG132 | Selleck | Cat#S2619 |
| Chloroquine (CQ) | Sigma | Cat#C6628 |
| Bafilomycin A1 (BAFA1) | Selleck | Cat#S1413 |
| cycloheximide（CHX） | Sigma | Cat#C7698 |
| Z-VAD-FMK | Selleck | Cat#S7023 |
| Salubrinal | Selleck | Cat#S2923 |
| ISRIB | MCE | Cat#HY-12495 |
| 4μ8C | Selleck | Cat#S7272 |
| Doxorubicin | Sigma | Cat#D1515 |
| Flag-Tag (Rabbit) | Sigma | Cat#F7425 |
| GADPH (Rabbit) | Proteintech | Cat#10494-1-AP |
| TRIP13 (Rabbit) | Proteintech | Cat#19602-1-AP |
| P-Akt(S473) (Rabbit) | Cell Signaling Technology | Cat#4060S |
| Akt (Rabbit) | Cell Signaling Technology | Cat#9272S |
| Actice MAPK (Rabbit) | Promega | Cat#V803A |
| ERK1/2 (Mouse) | Santa Cruz | Cat#Sc-514302 |
| Ki67 | Cell Signaling Technology | Cat#9449 |
| CyclinD1 | Santa Cruz | Cat#sc-20044 |
| caspase-3 | Cell Signaling Technology | Cat#9662S |
| PARP | Cell Signaling Technology | Cat#9532S |
| Ubiquitin(P4G7) | Convance | Cat#MMS-258R |
| CHOP | Cell Signaling Technology | Cat#2895S |
| Bip | Cell Signaling Technology | Cat#3177S |
| ATF4 | Cell Signaling Technology | Cat#11815S |
| P-PRAS40(T246) | Cell Signaling Technology | Cat#2997S |
| P62 | Sigma | Cat#P0067 |
| LC3 | Sigma | Cat#L7543 |
