# Supplementary material for: Osimertinib induces paraptosis and TRIP13 confers resistance in glioblastoma cells
Source: Cell Death Discov. 2023 Sep 5;9:333. doi: 10.1038/s41420-023-01632-6 (PMC10480197; doi:10.1038/s41420-023-01632-6)
Supplement: Supplementary file 3 — Original Data File [file 41420_2023_1632_MOESM3_ESM.docx]

Fig1E:

LN-229: CyclinD1 GAPDH U87MG: CyclinD1 GAPDH


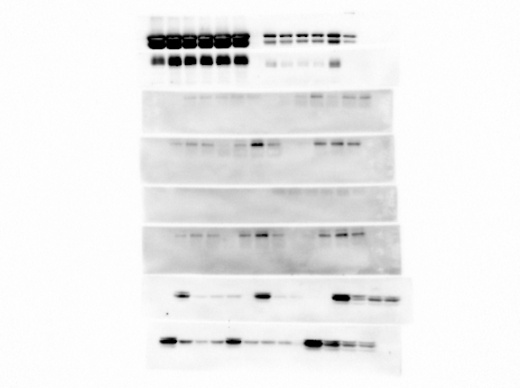

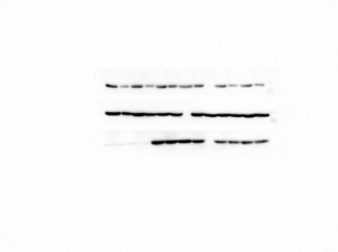

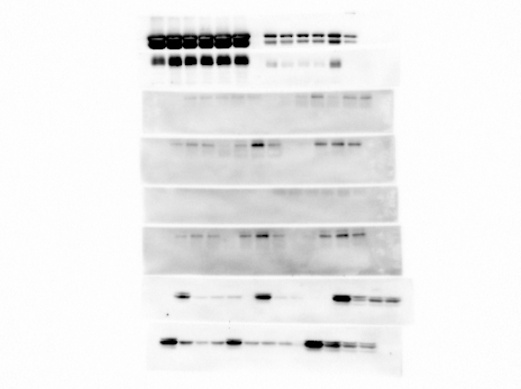

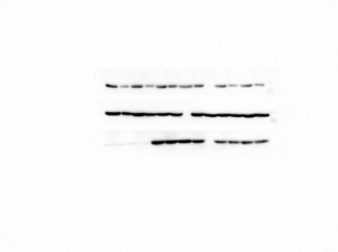


Fig1F:

LN-229: CyclinD1 GAPDH U87MG: CyclinD1 GAPDH


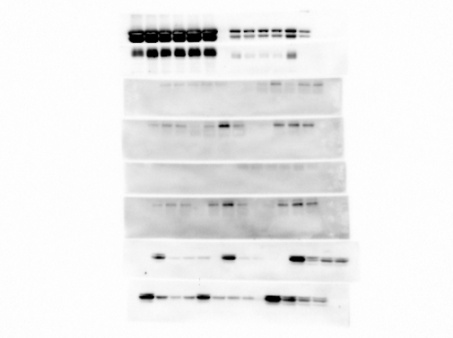

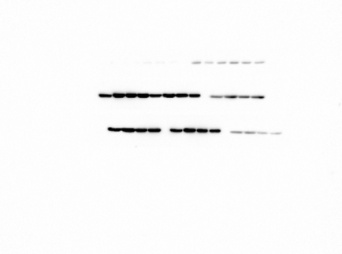

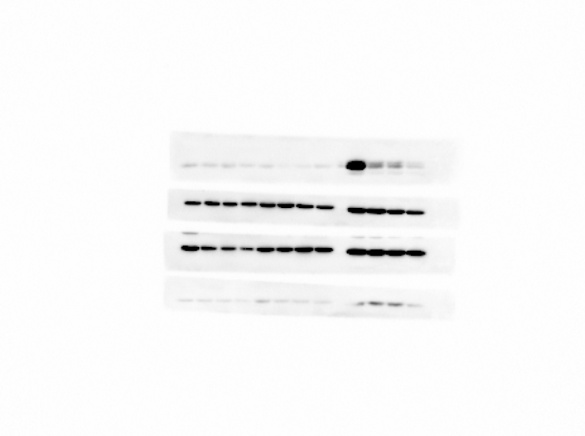

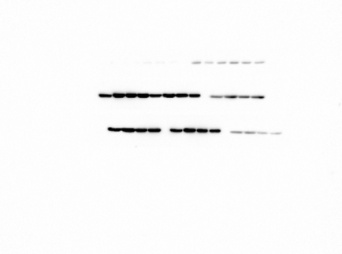


Fig1G:

LN-229: P-AKT(S473) AKT P-ERK1/2 ERK1/2 GAPDH


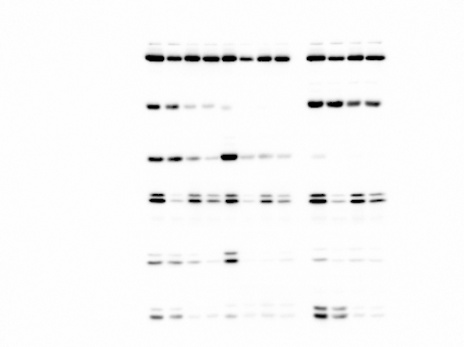

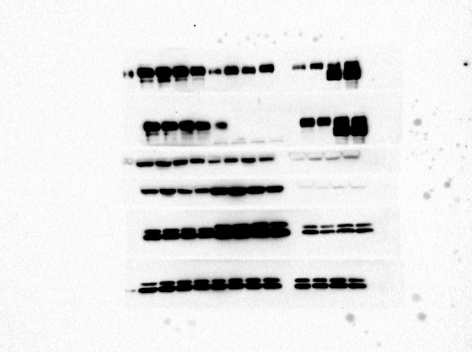

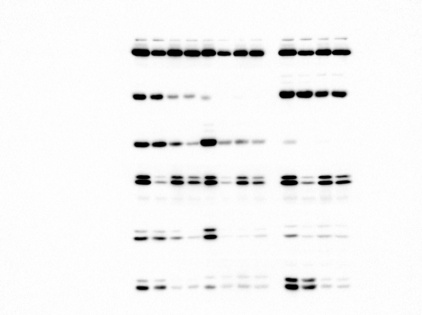

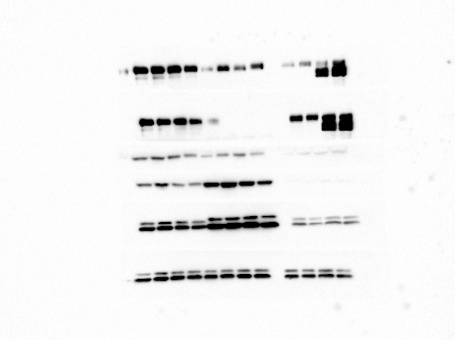

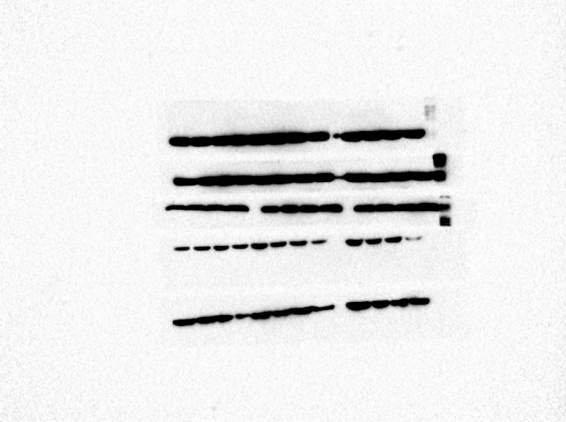


U87MG: P-AKT(S473) AKT P-ERK1/2 ERK1/2 GAPDH


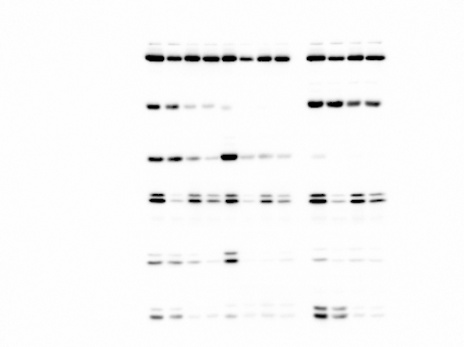

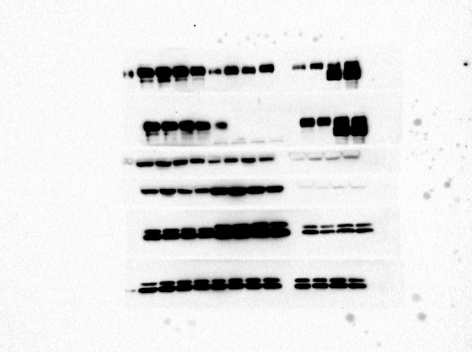

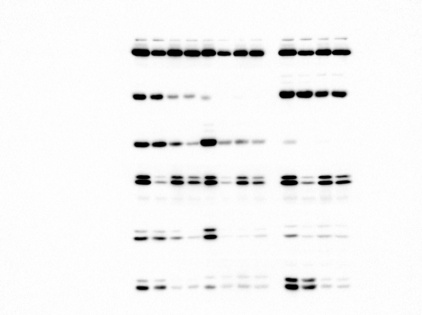

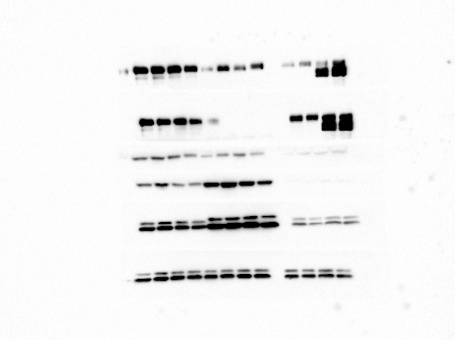

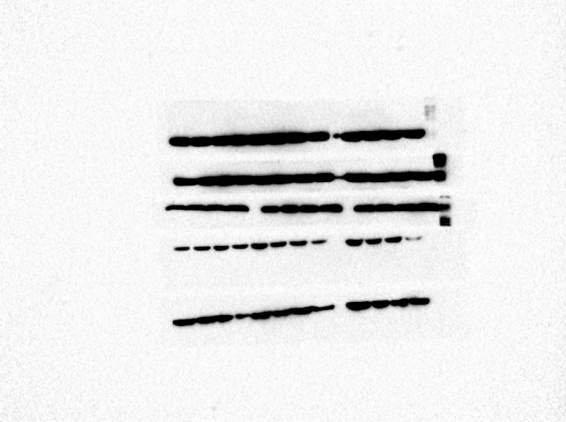


Fig2A:

LN-229:caspase-3 PARP GAPDH


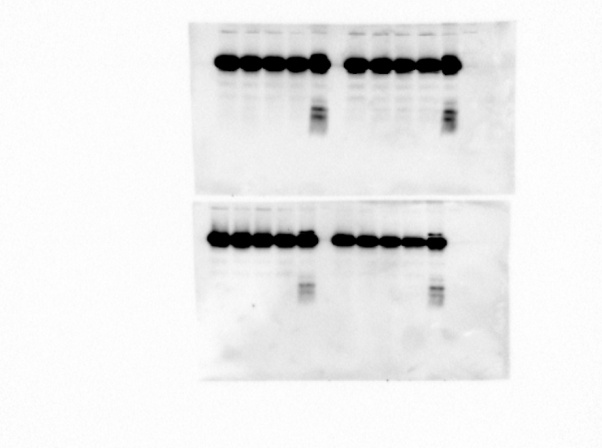

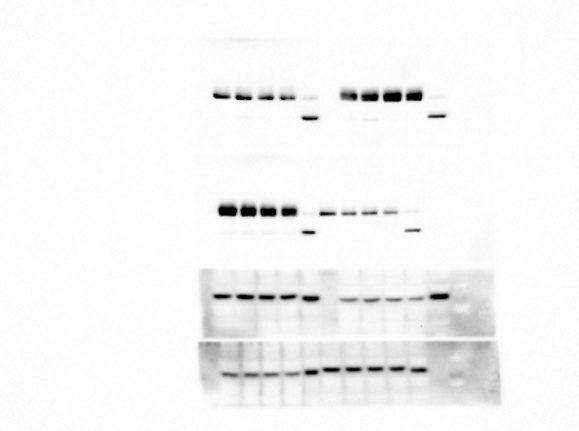

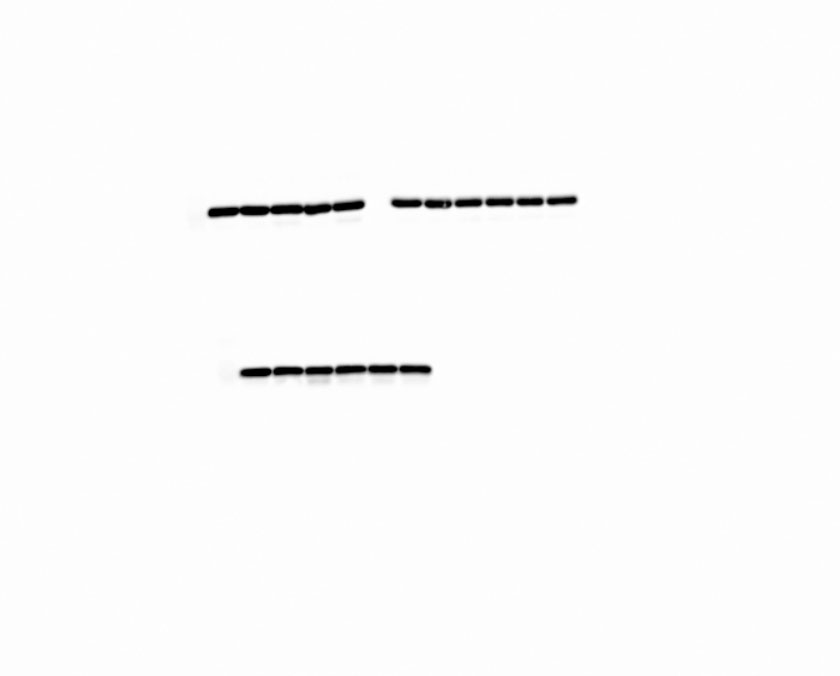


U87MG:caspase-3 PARP GAPDH


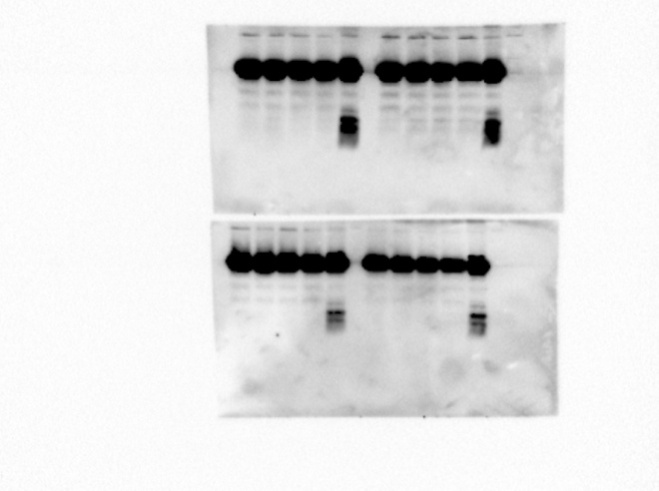

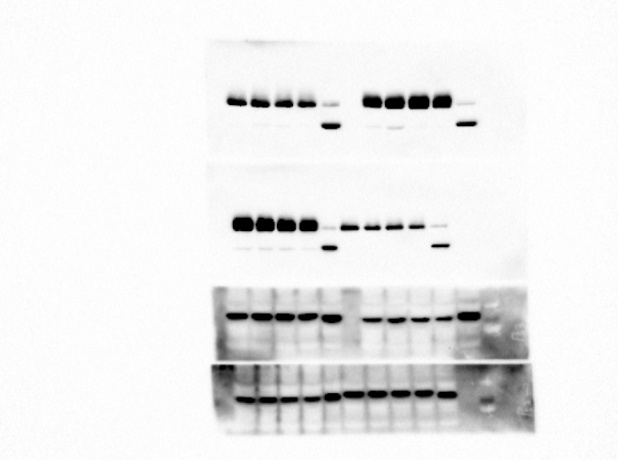

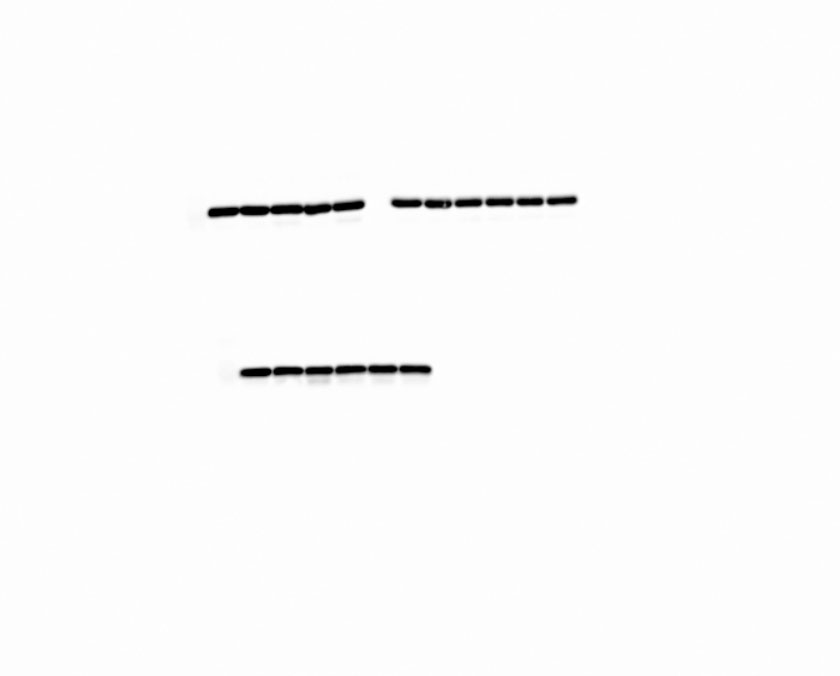


Fig2B:

LN-229:caspase-3 PARP GAPDH


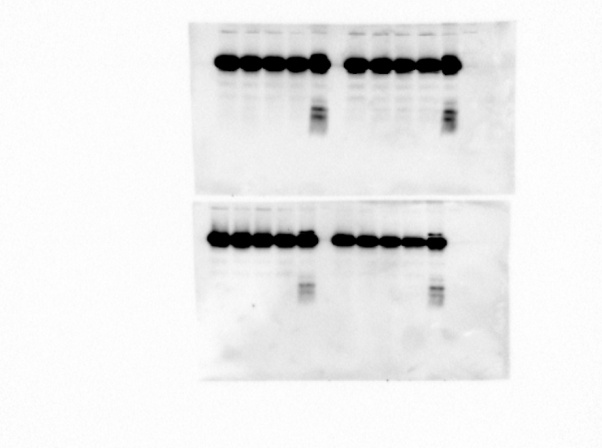

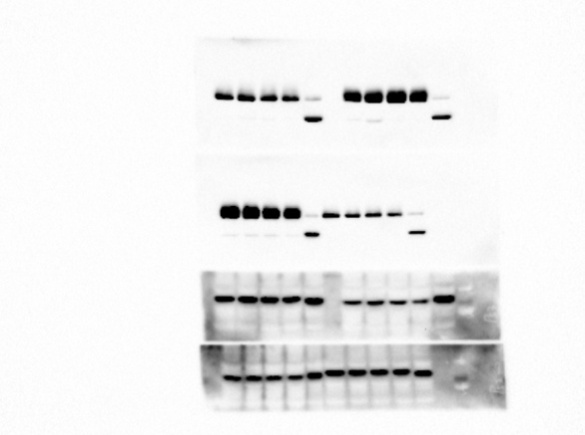

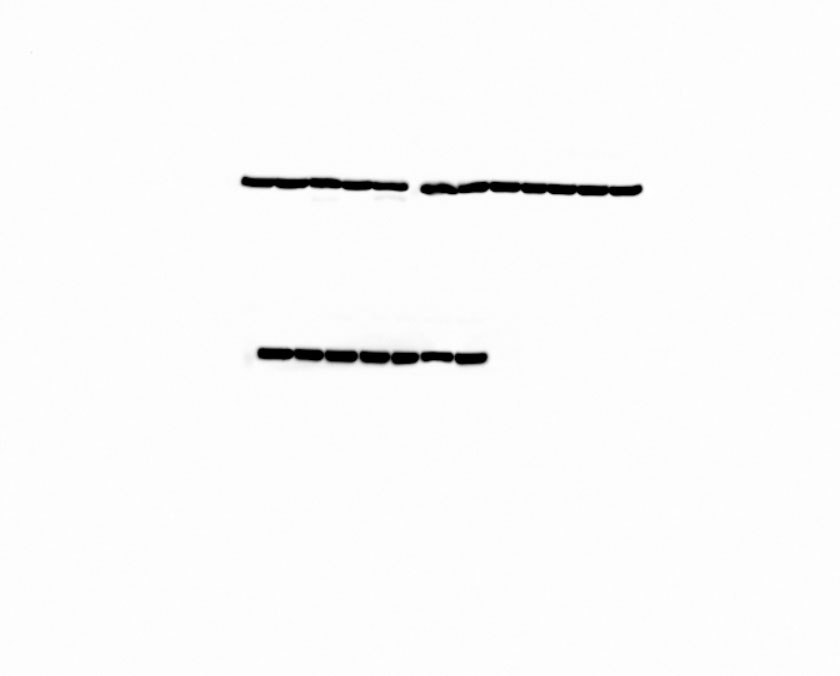


U87MG:caspase-3 PARP GAPDH


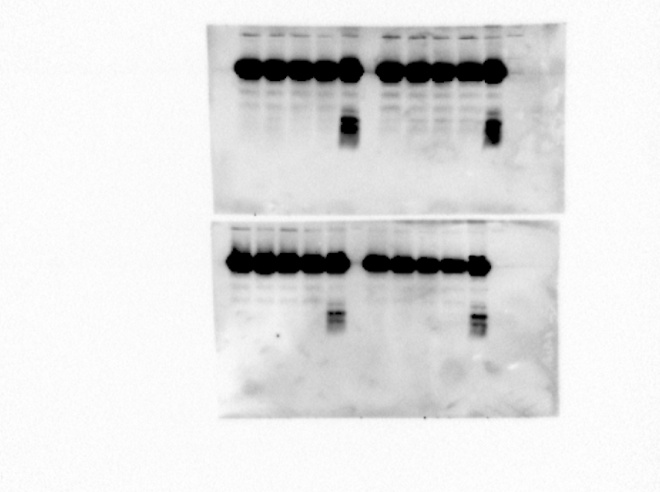

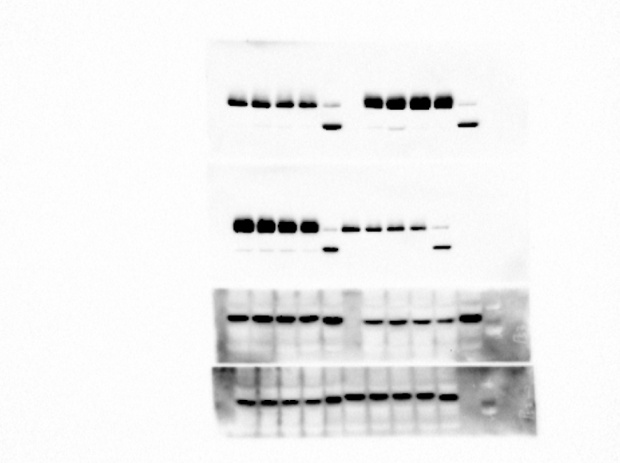

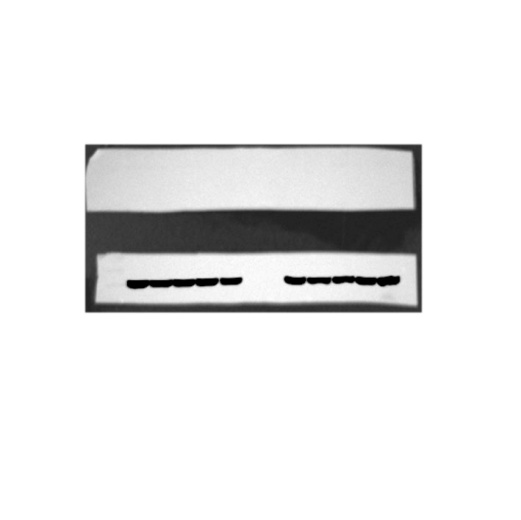


Fig2F:

LN-229: Ub GAPDH U87MG: Ub GAPDH


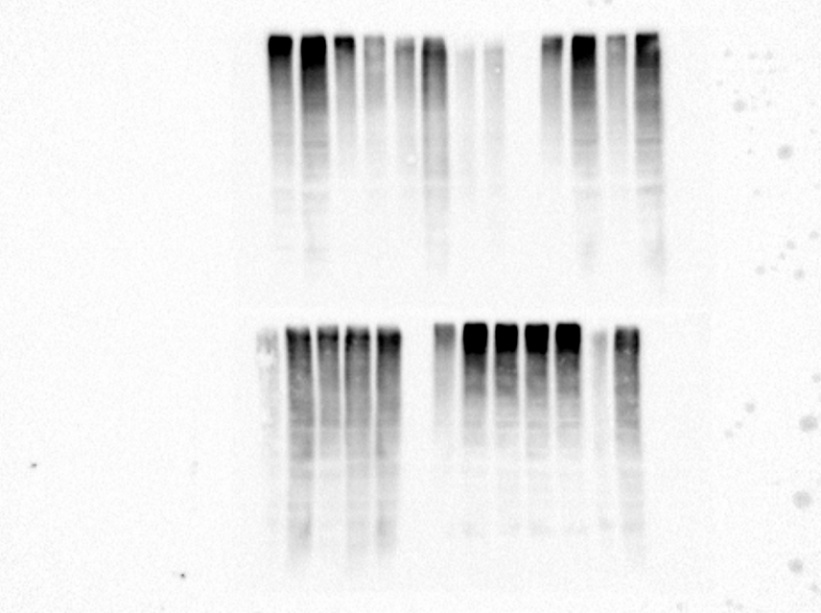

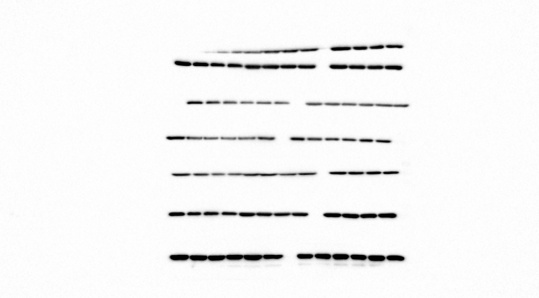

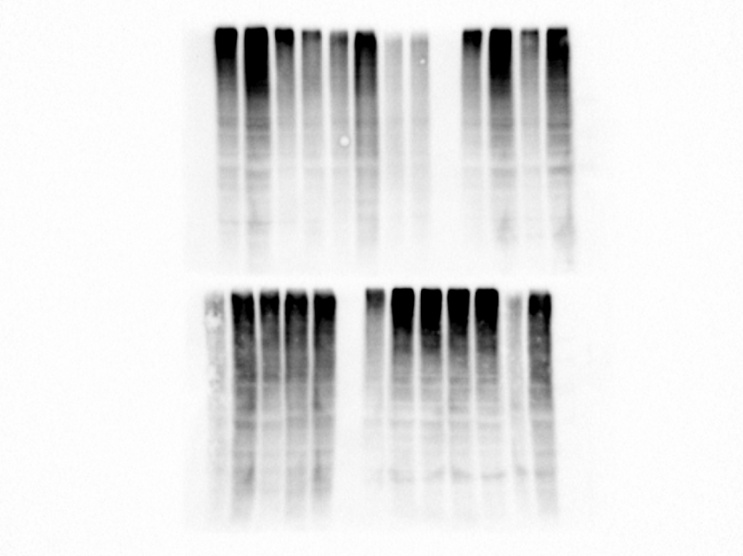

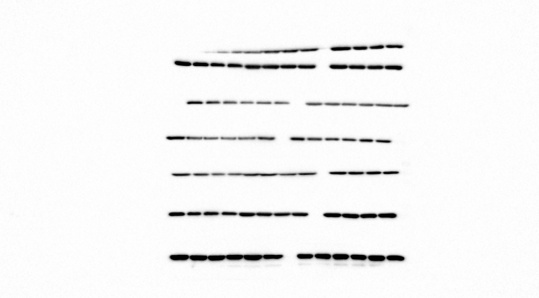


Fig2H:

LN-229 CHOP Bip ATF4 GAPDH


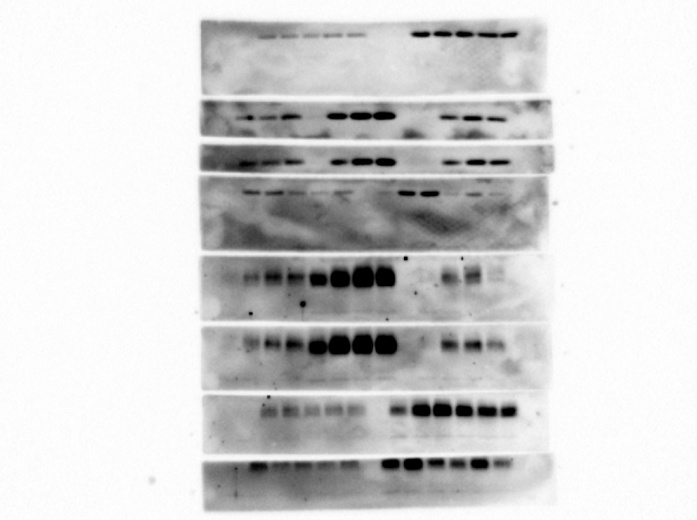

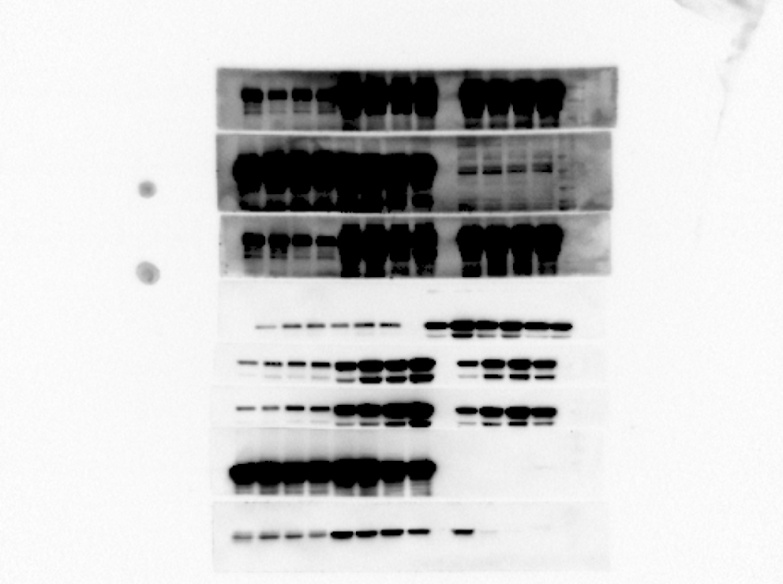

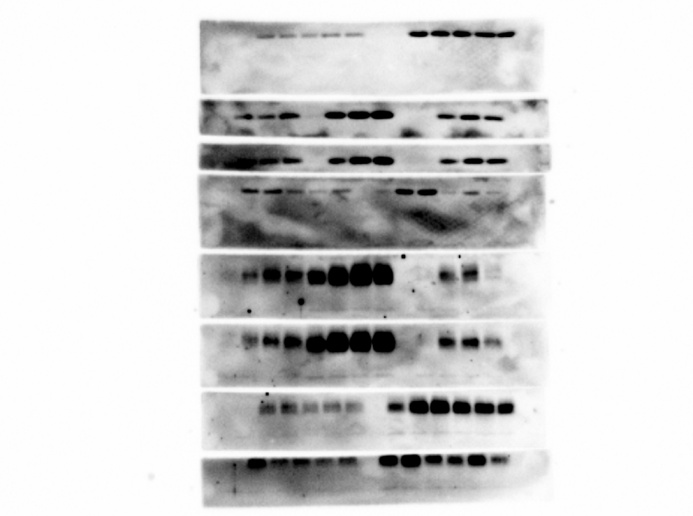

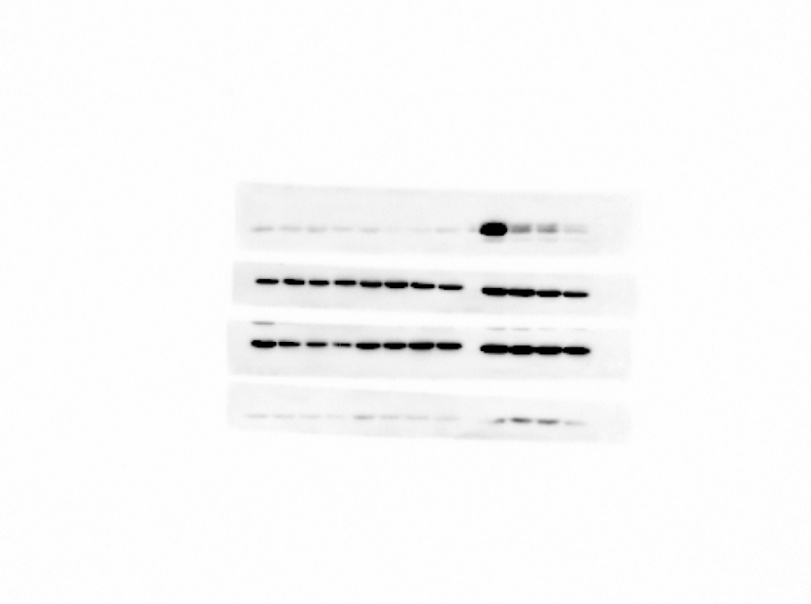


U87MG CHOP Bip ATF4 GAPDH


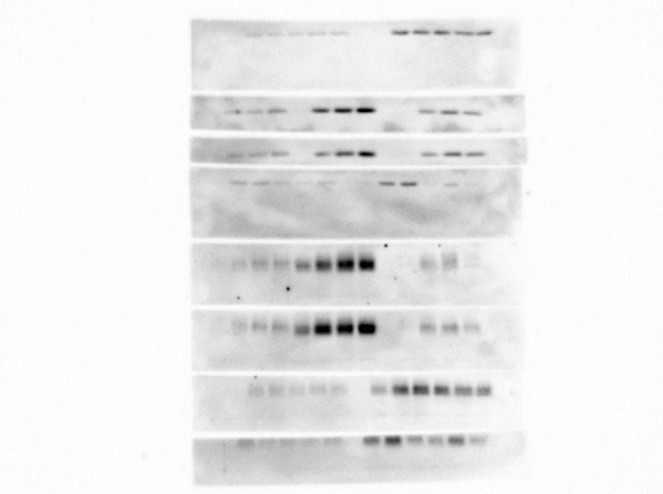

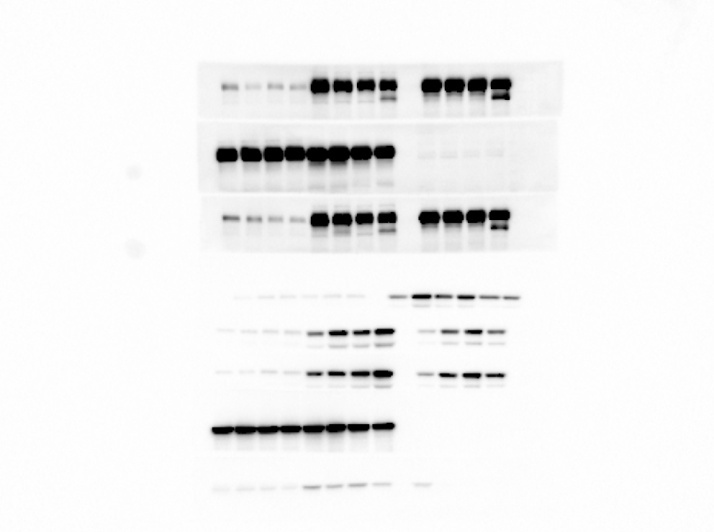

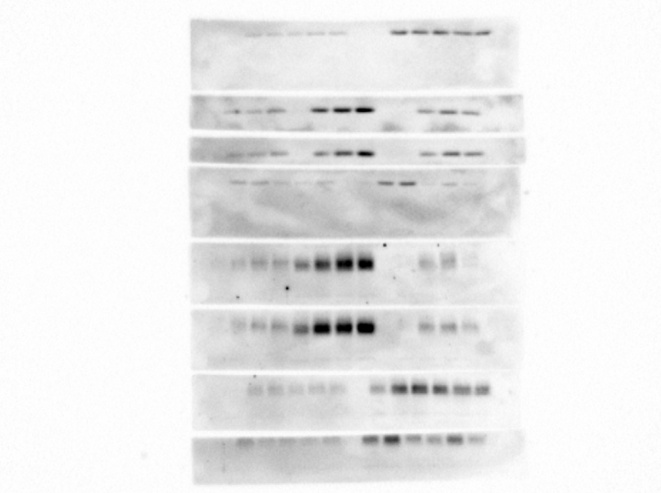

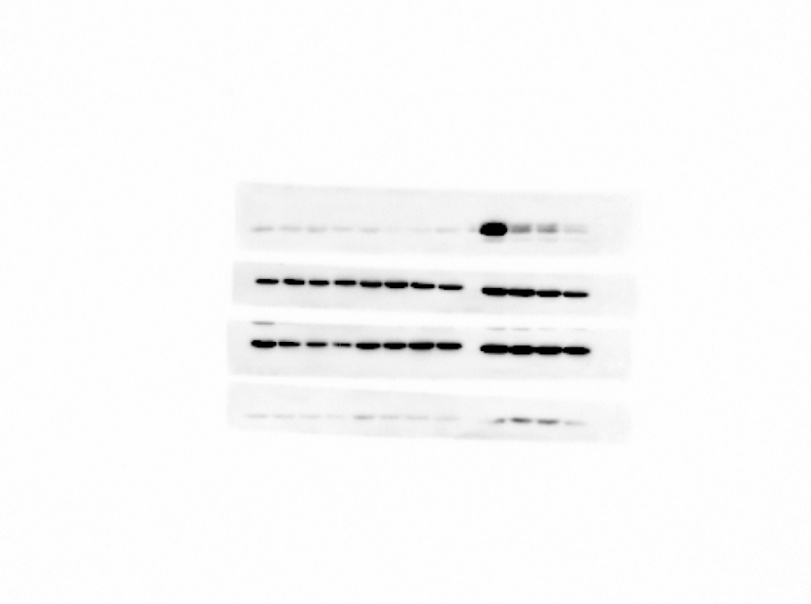


Fig2I:

LN-229 CHOP Bip ATF4 GAPDH


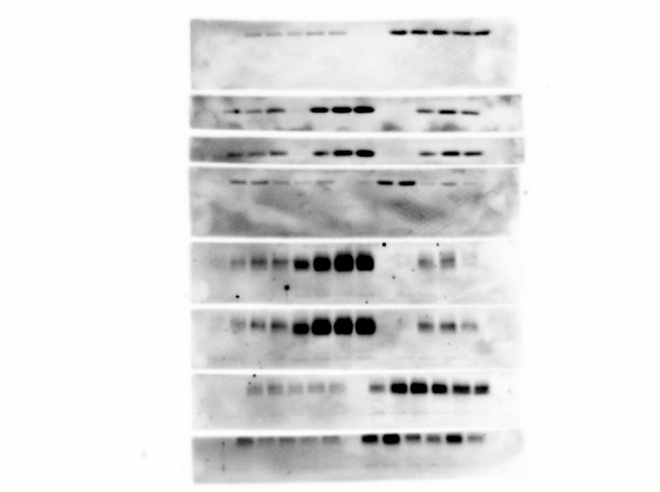

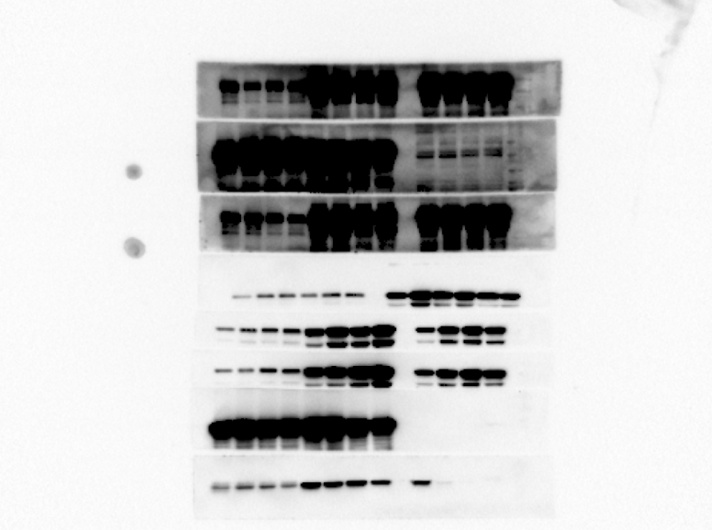

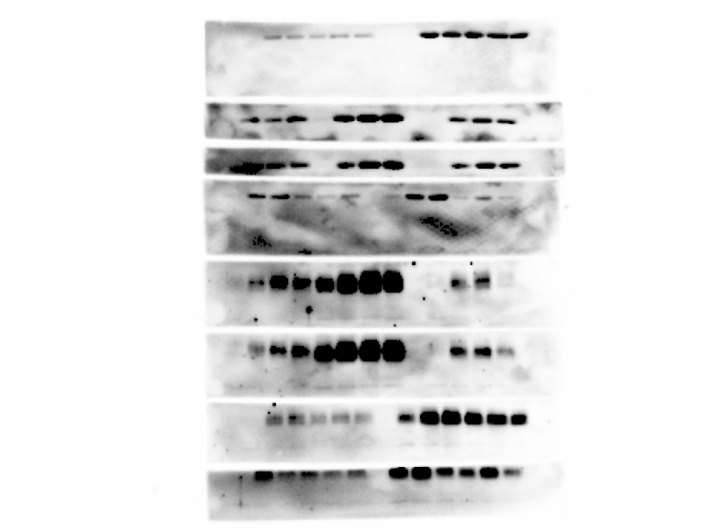

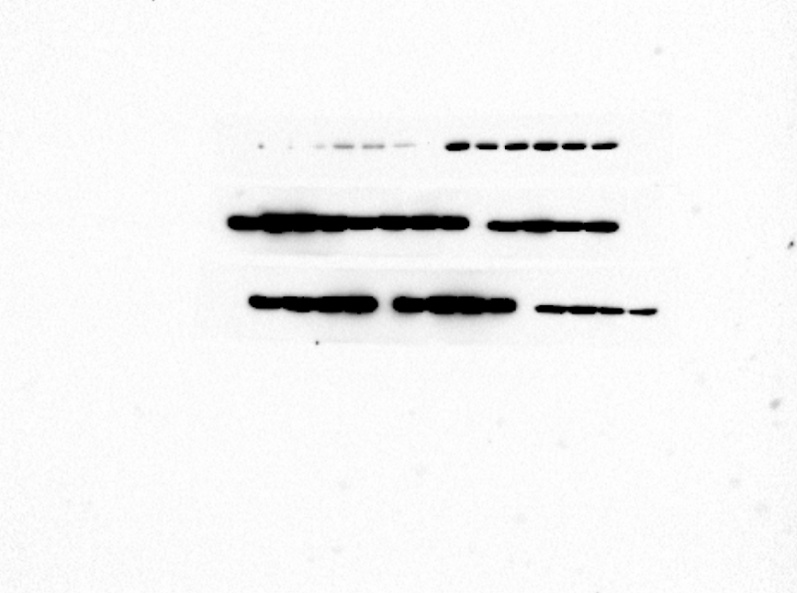


U87MG CHOP Bip ATF4 GAPDH


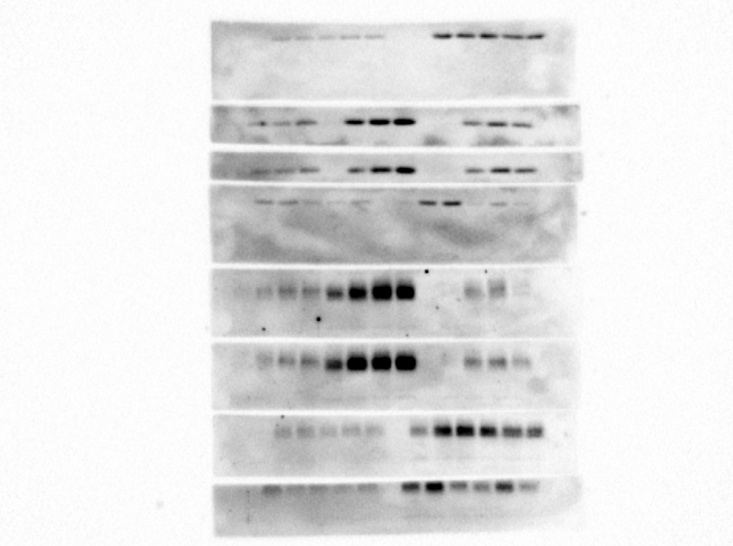

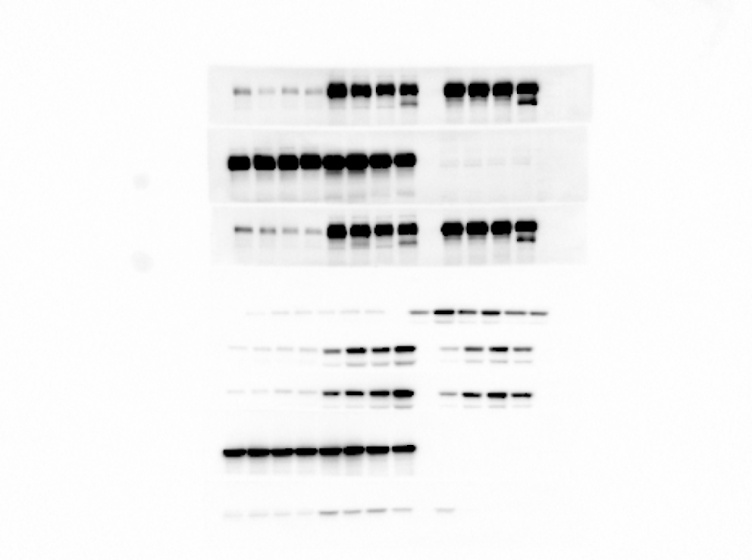

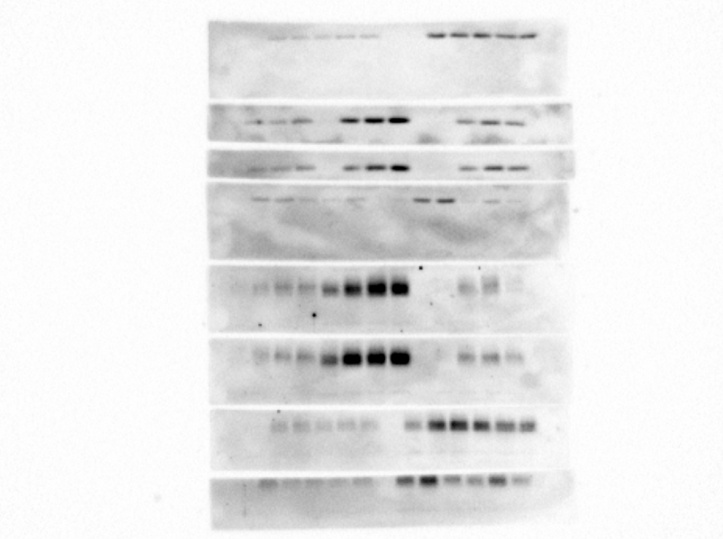

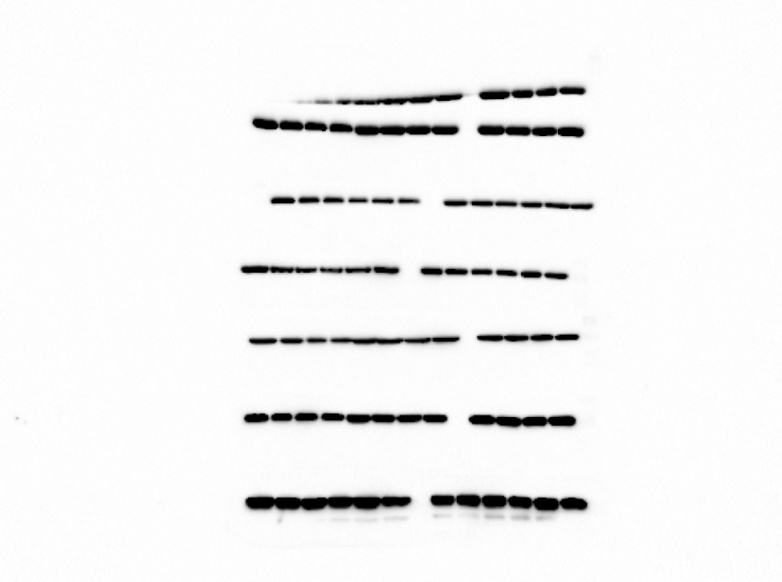


Fig2J:

LN-229 CHOP Bip GAPDH


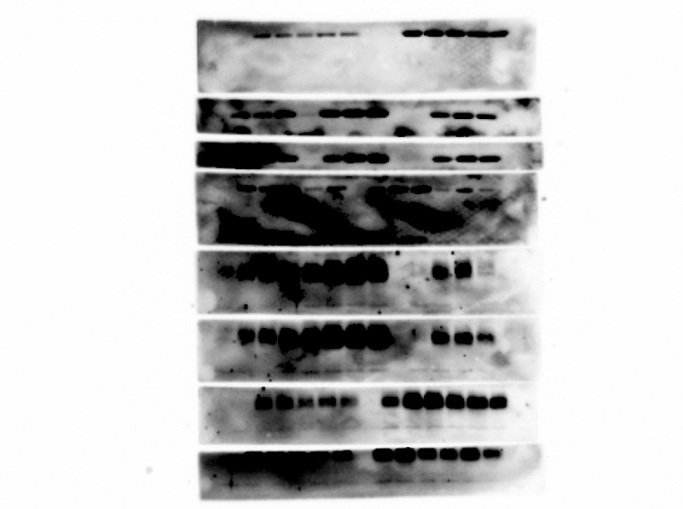

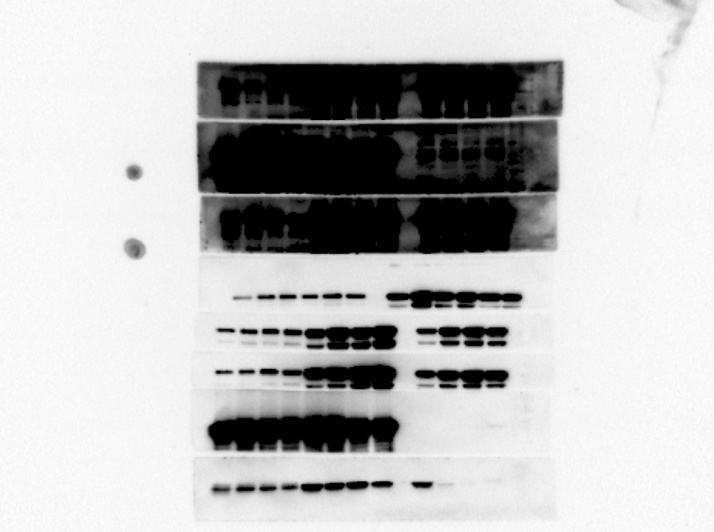

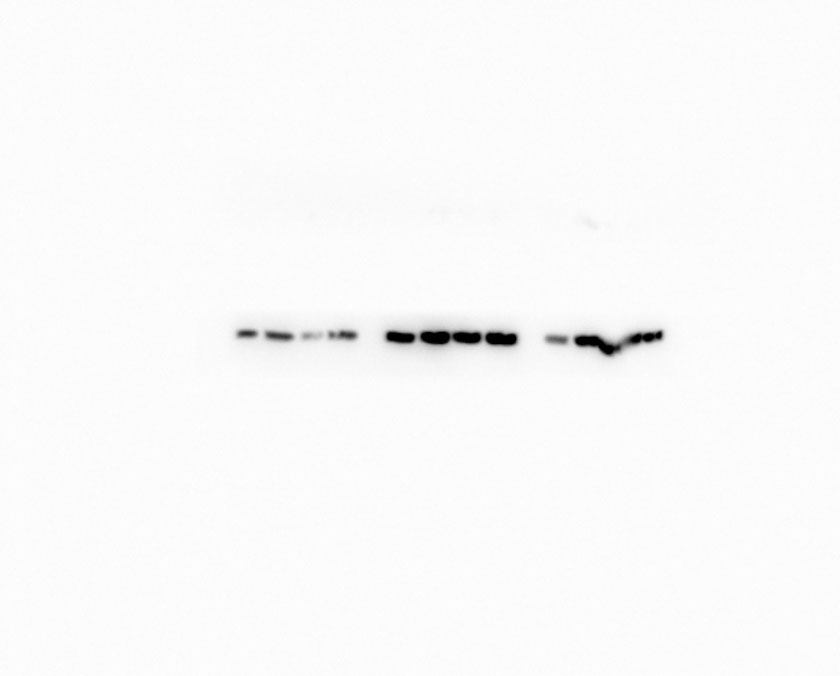


LN-229 CHOP Bip GAPDH


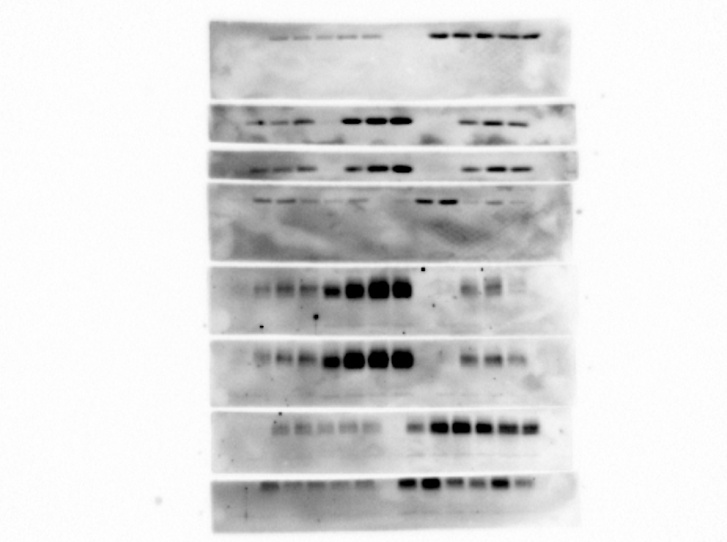

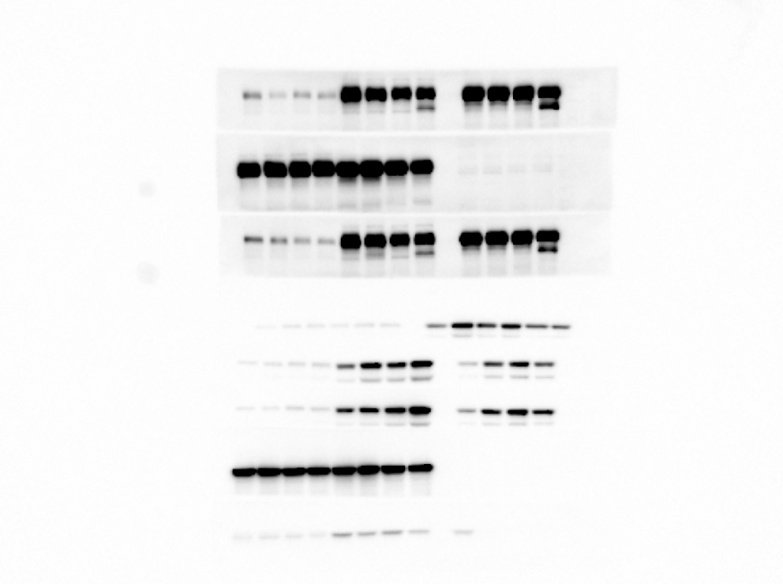

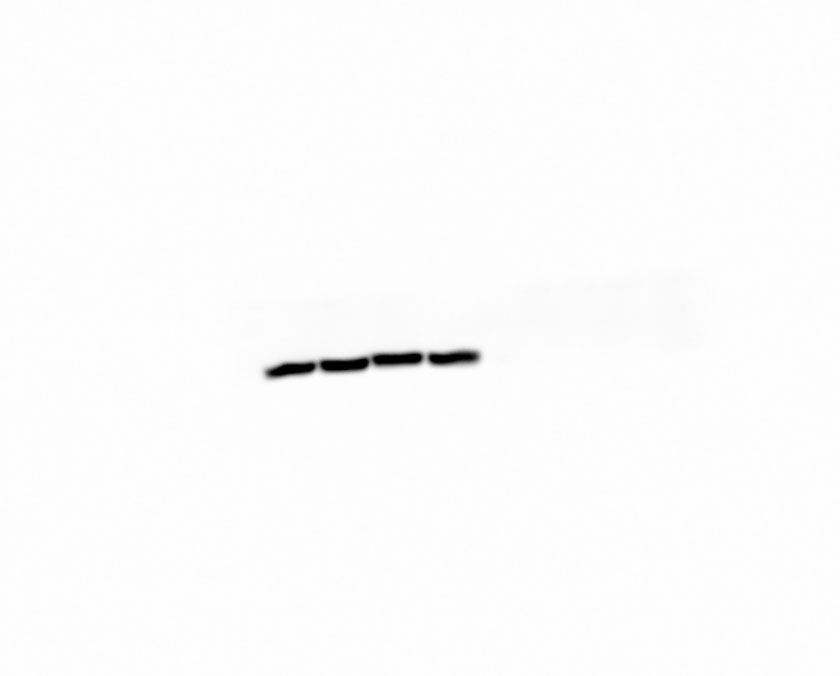


Fig3B:

LN-229: Ub CHOP GAPDH


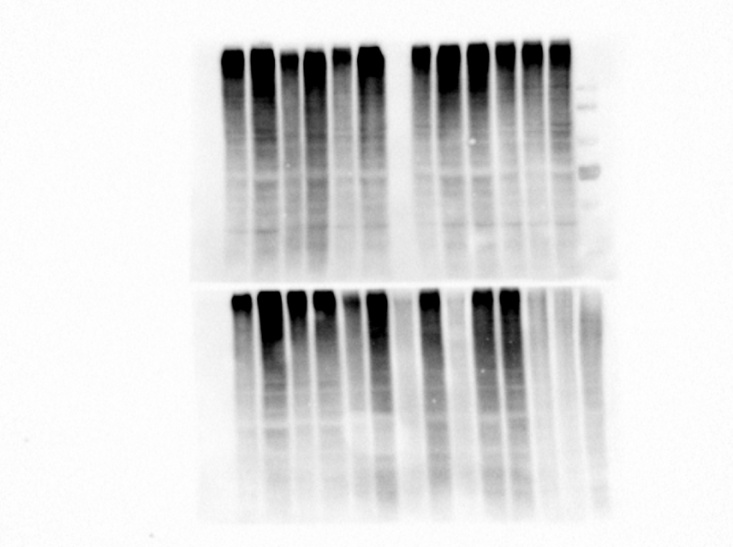

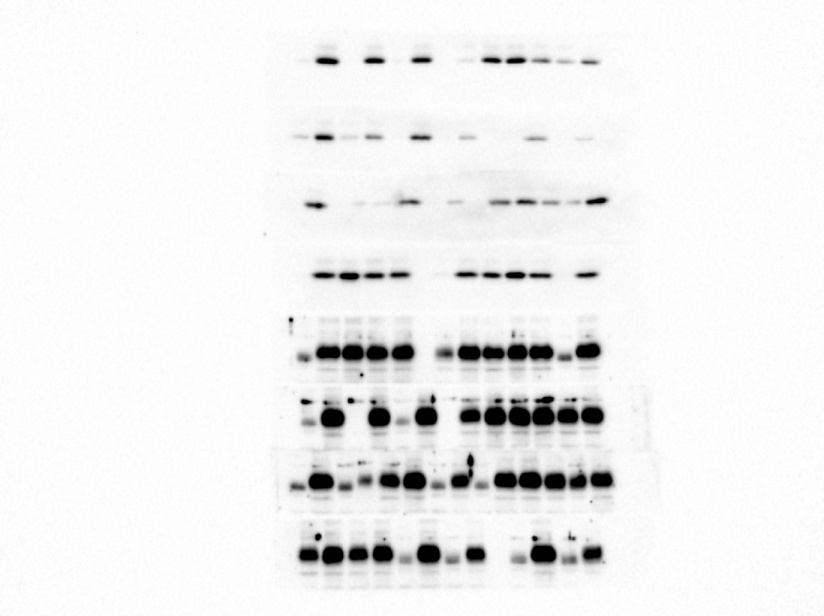

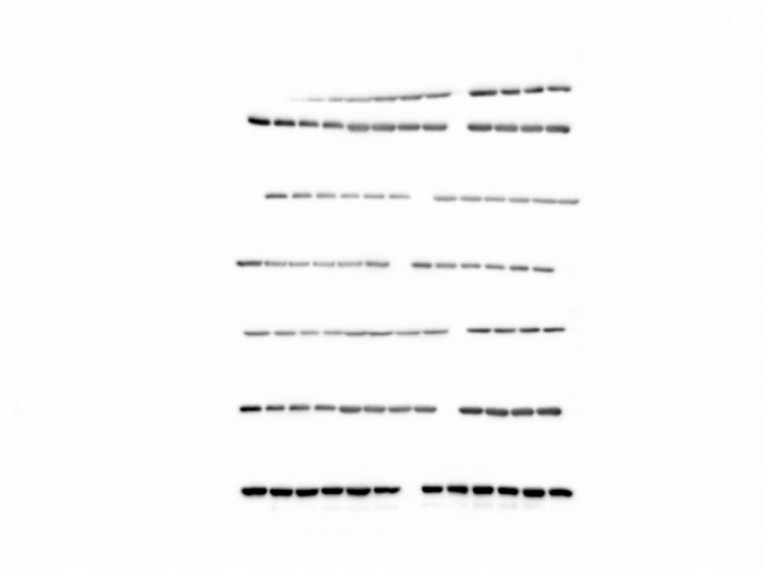


U87MG: Ub CHOP GAPDH


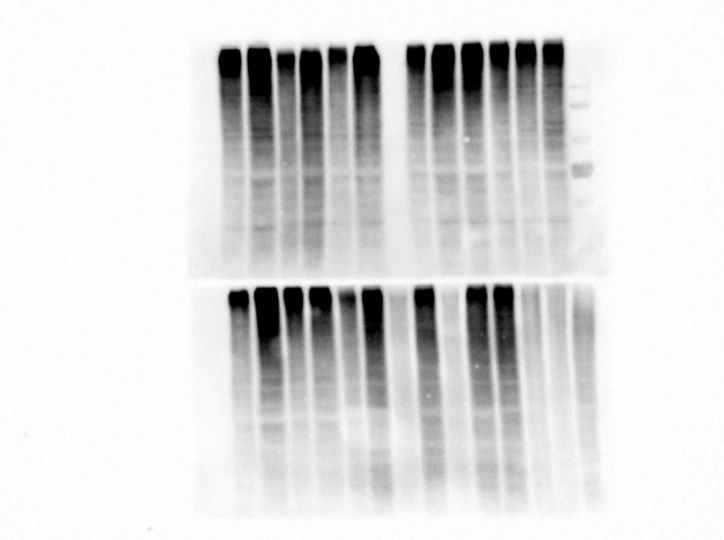

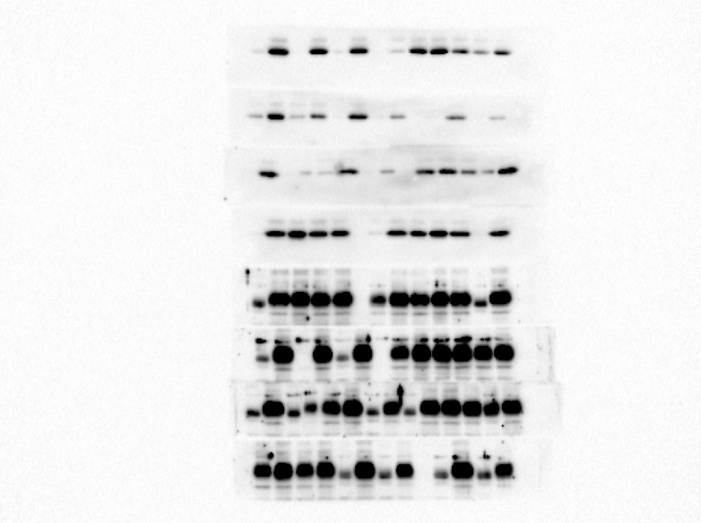

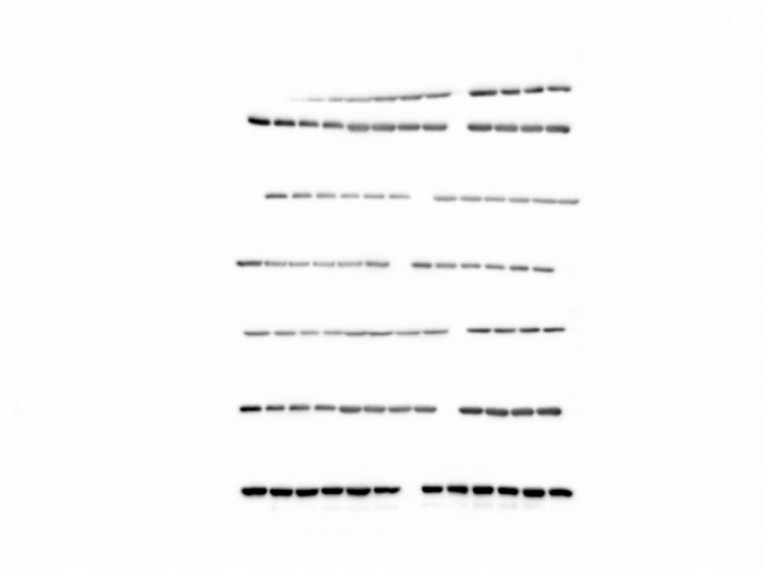


Fig3D:

LN-229: Ub CHOP GAPDH


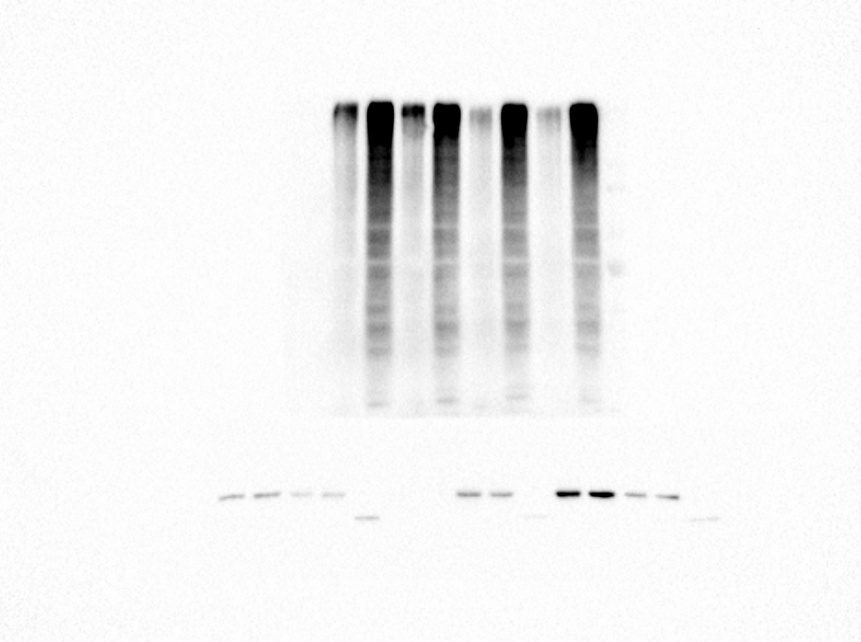

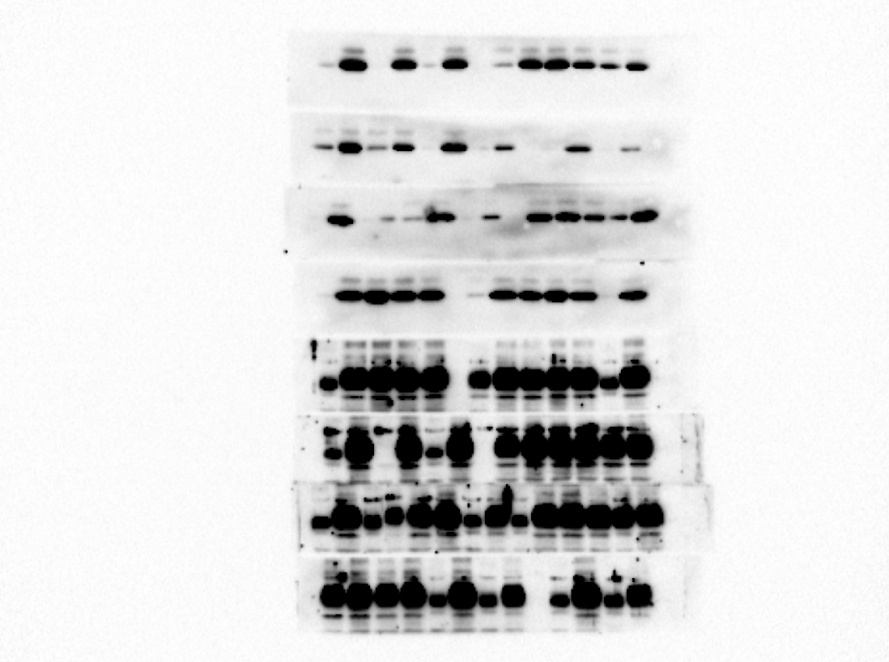

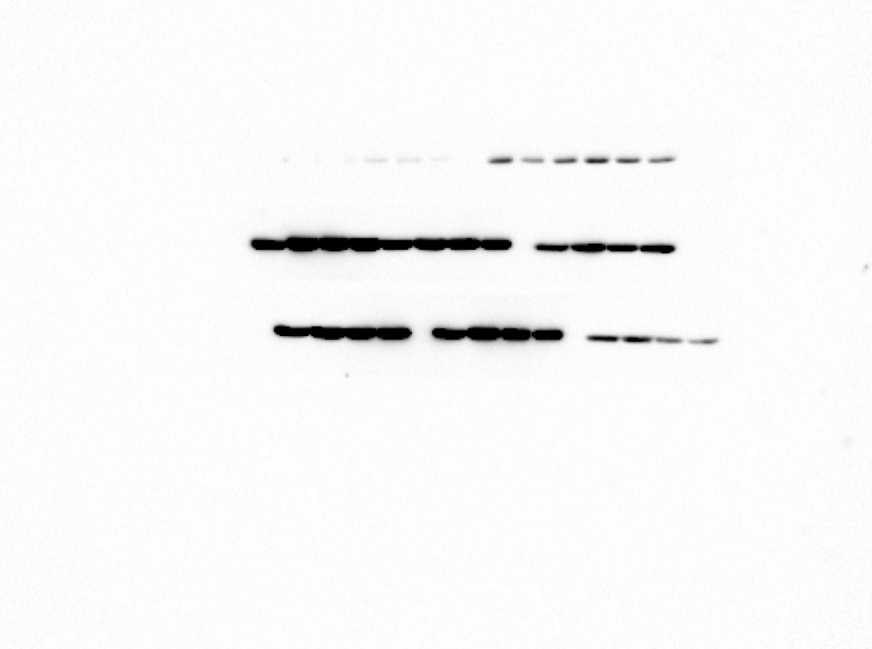


U87MG: Ub CHOP GAPDH


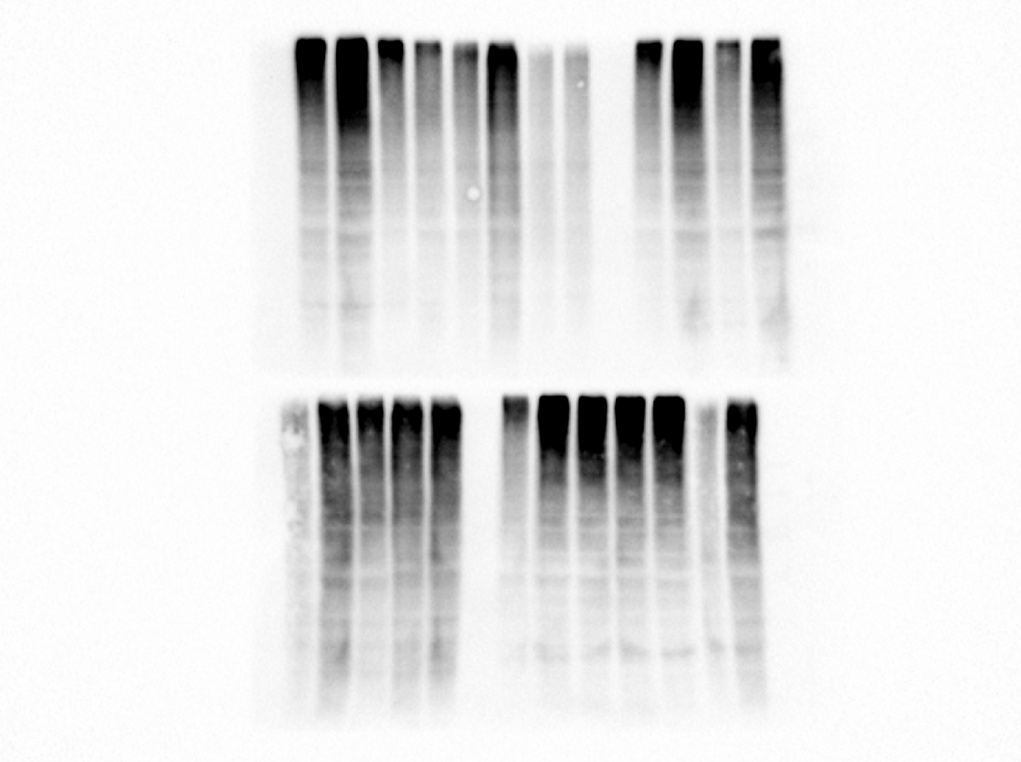

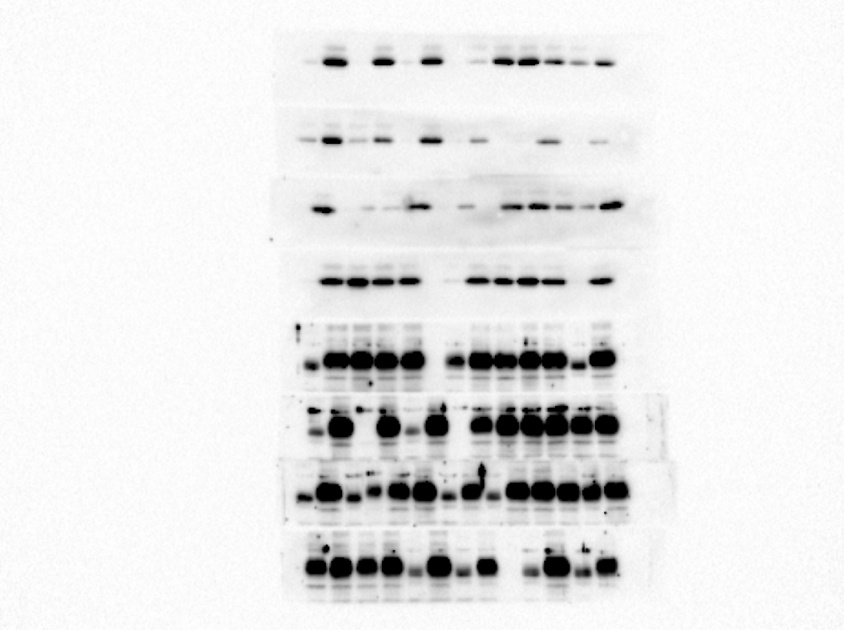

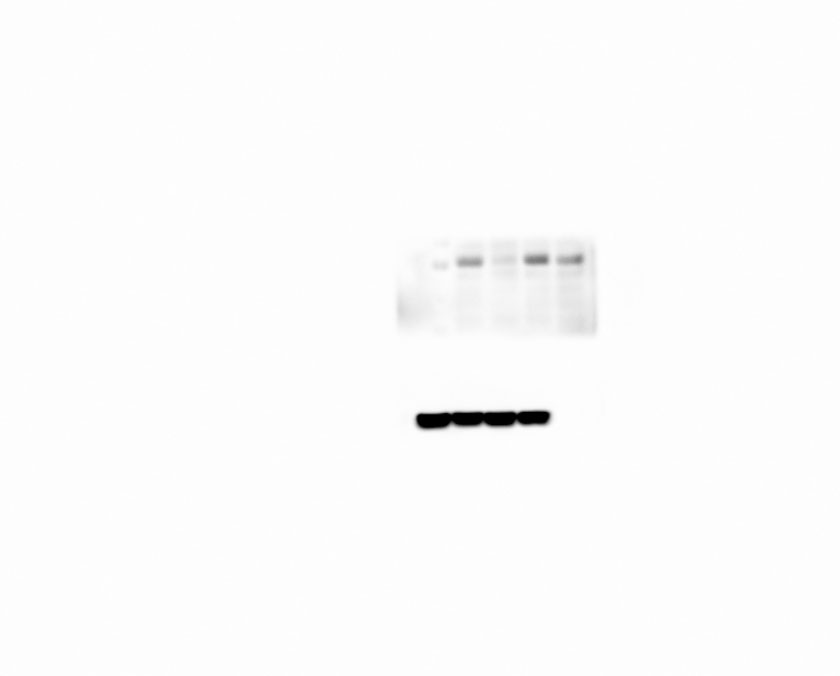


Fig4D:

LN-229: CHOP P-ERK1/2 TRIP13 Flag GAPDH


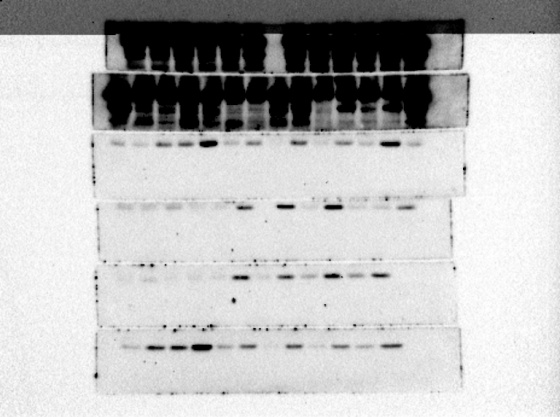

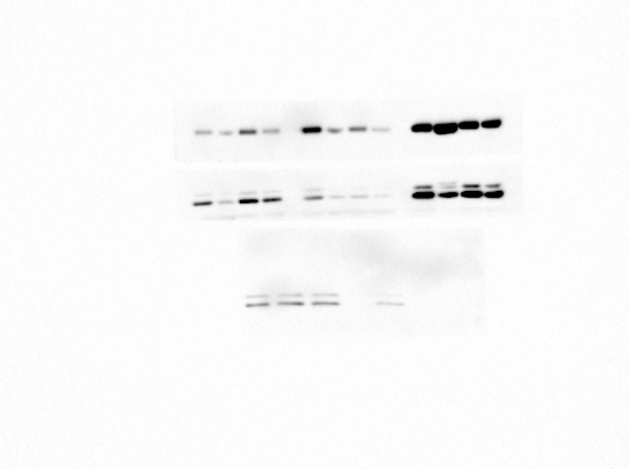

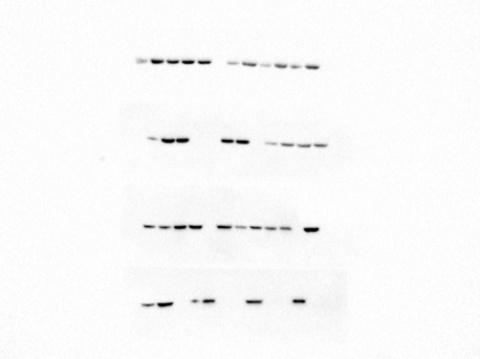

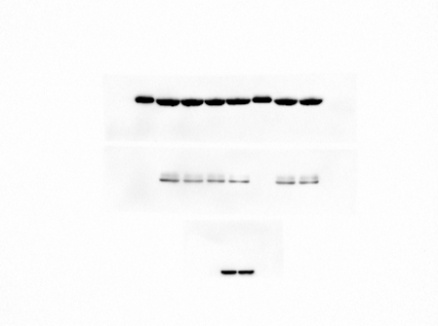

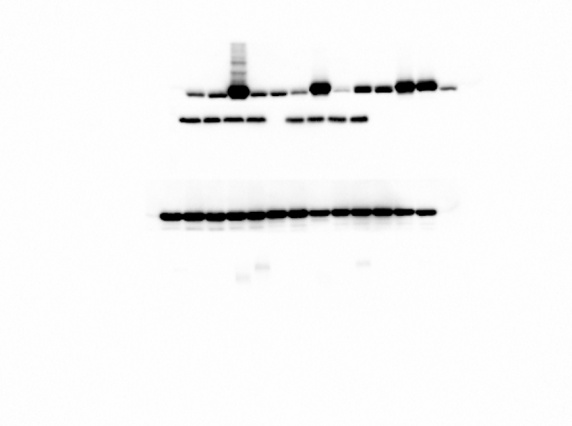


Fig4E:

U87MG: CHOP P-ERK1/2 TRIP13 GAPDH


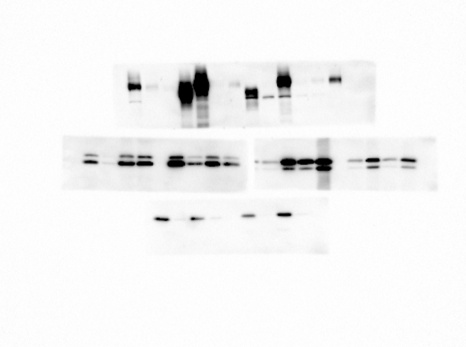

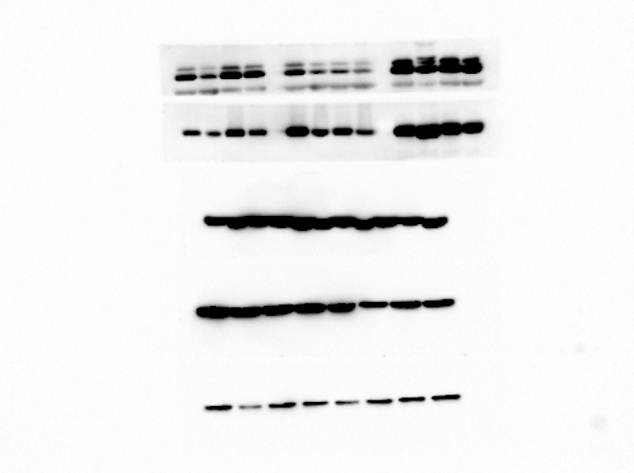

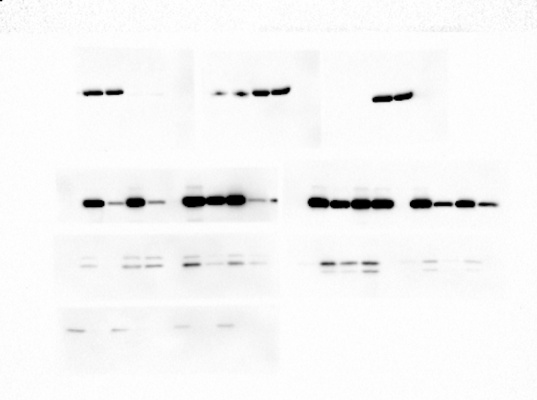

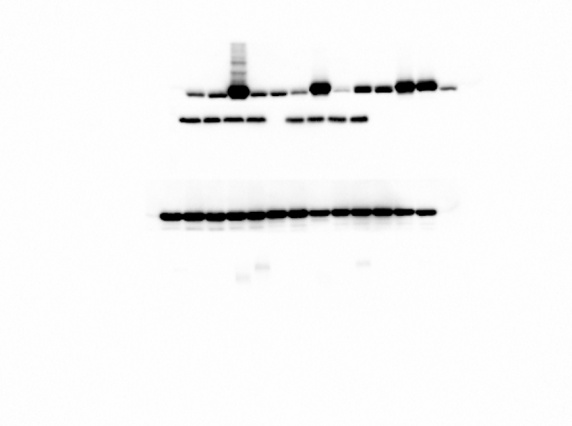


Fig5A:

LN-229: TRIP13(left) TRIP13(right) GAPDH(left) GAPDH(right)


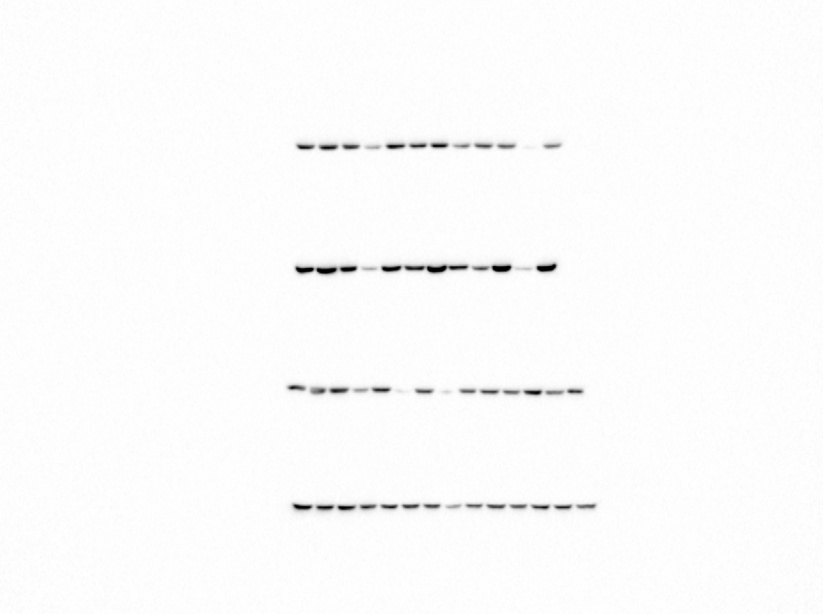

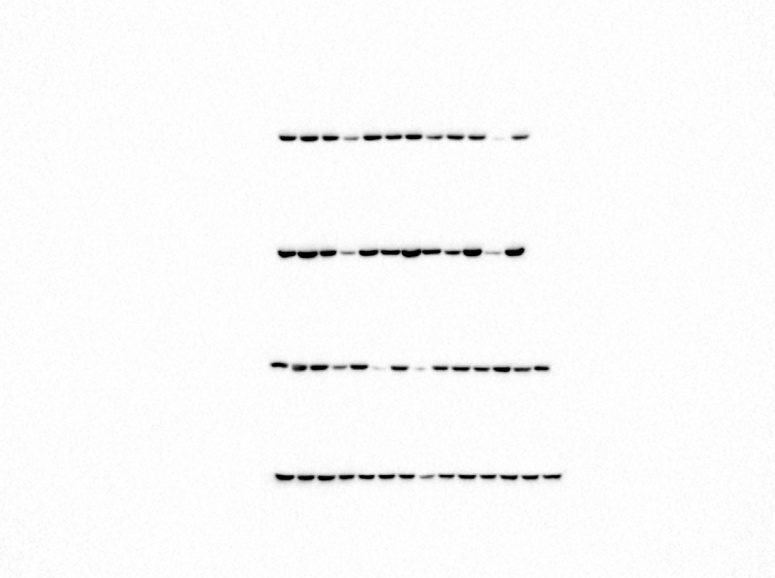

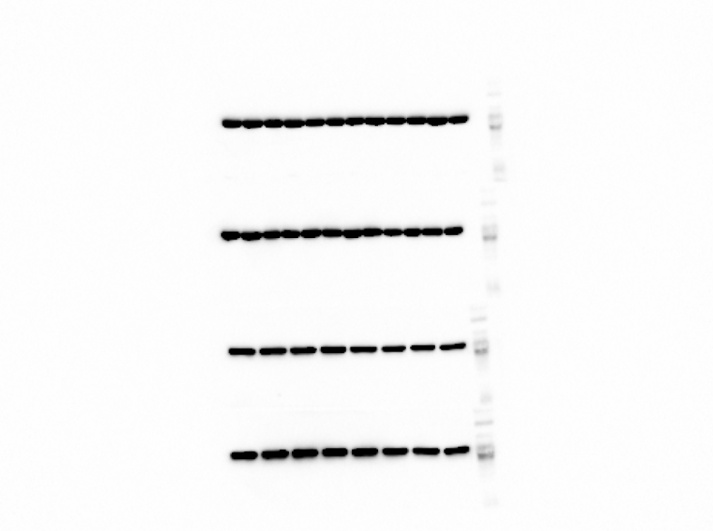

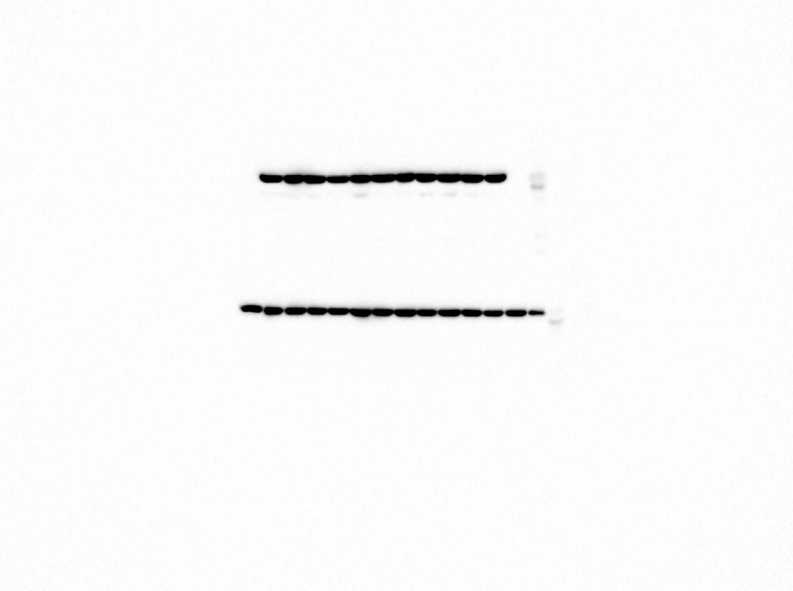


Fig5B:

U87MG: TRIP13(left) TRIP13(right) GAPDH(left) GAPDH(right)


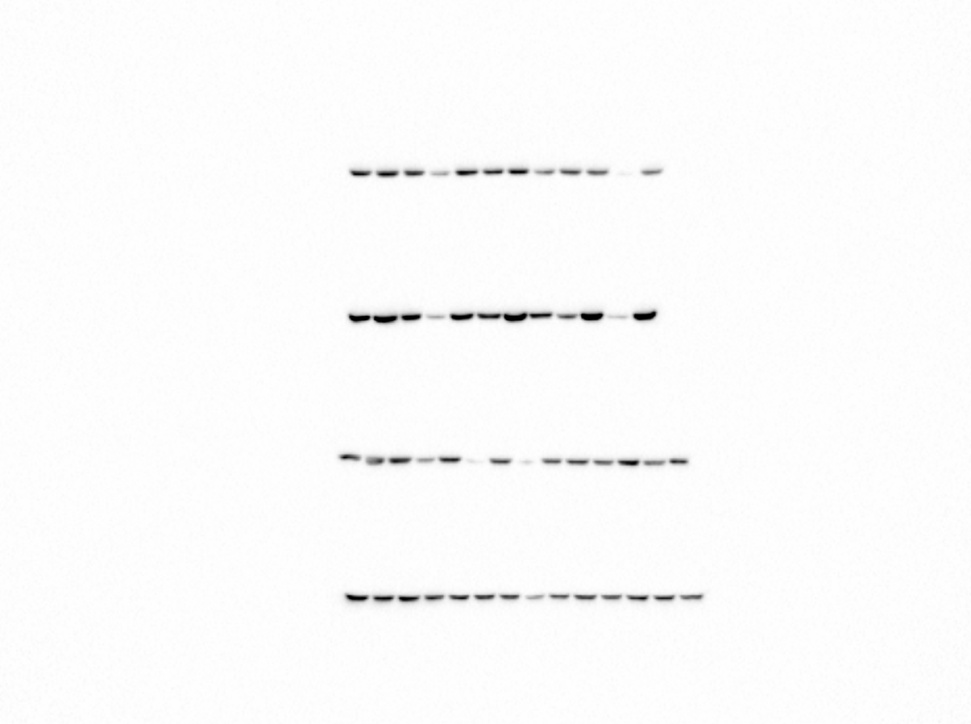

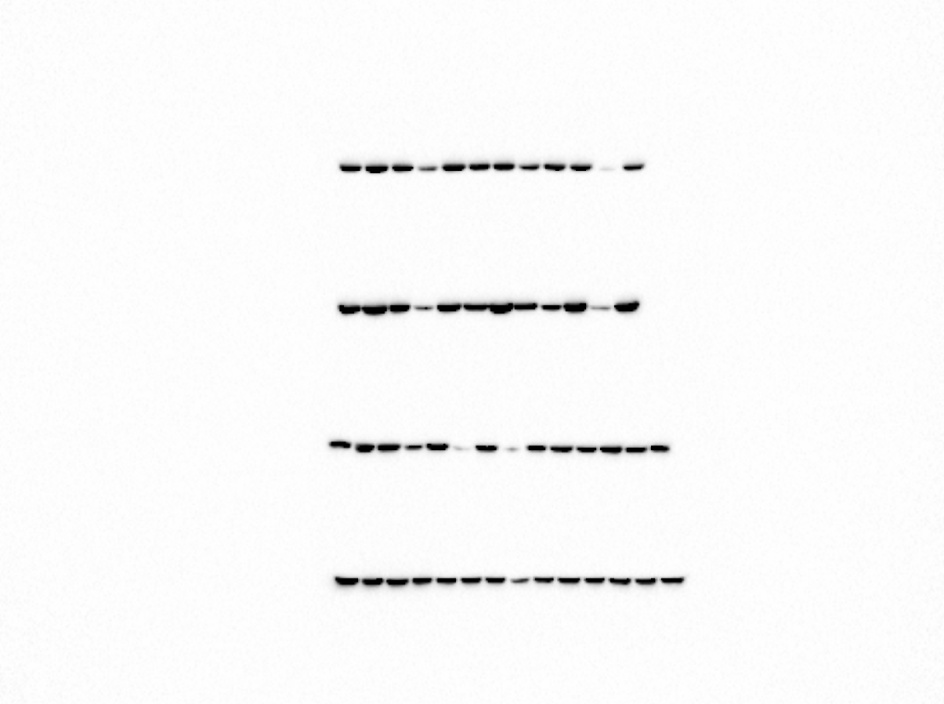

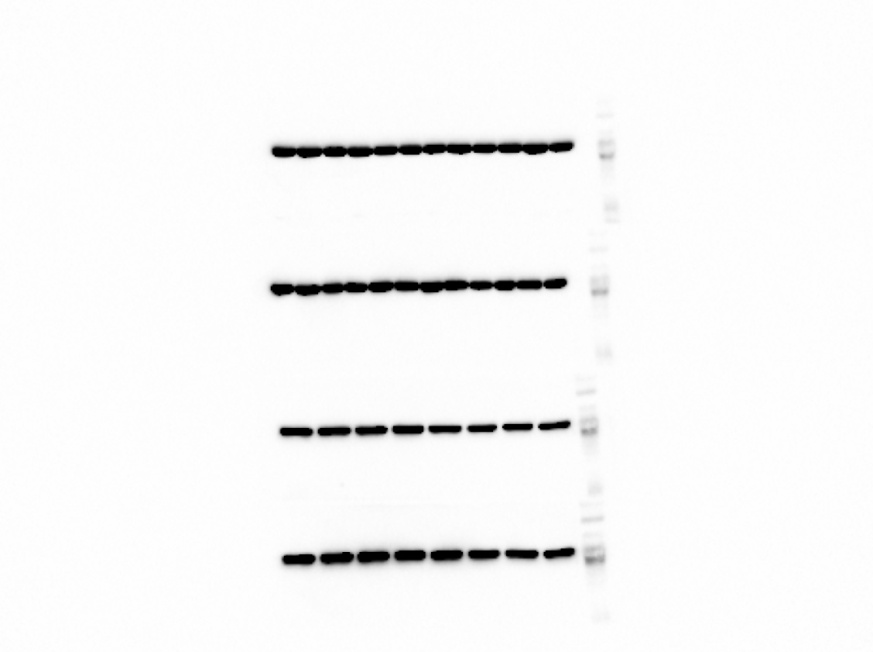

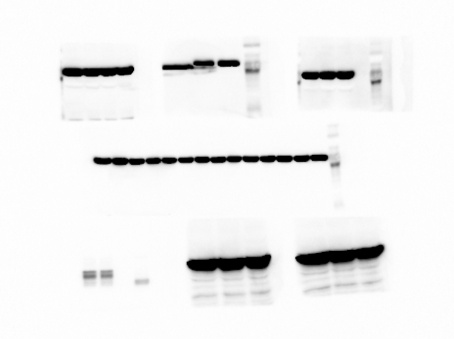


Fig5C:

LN-229: P-AKT(S473) P-PRAS40(T246) GAPDH


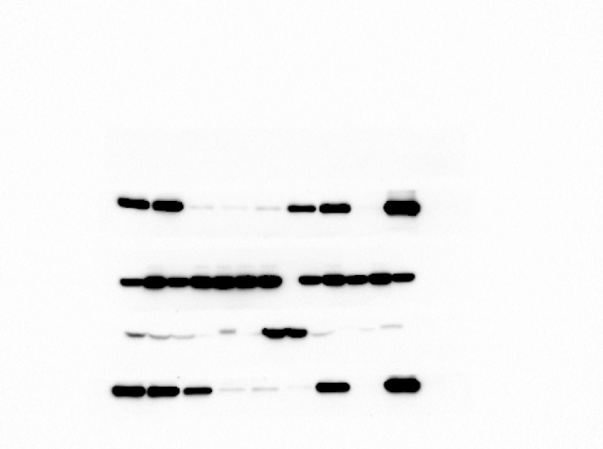

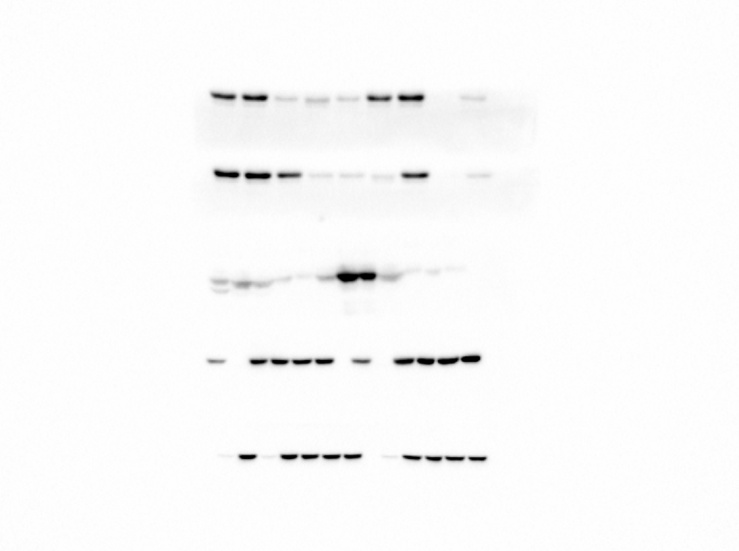

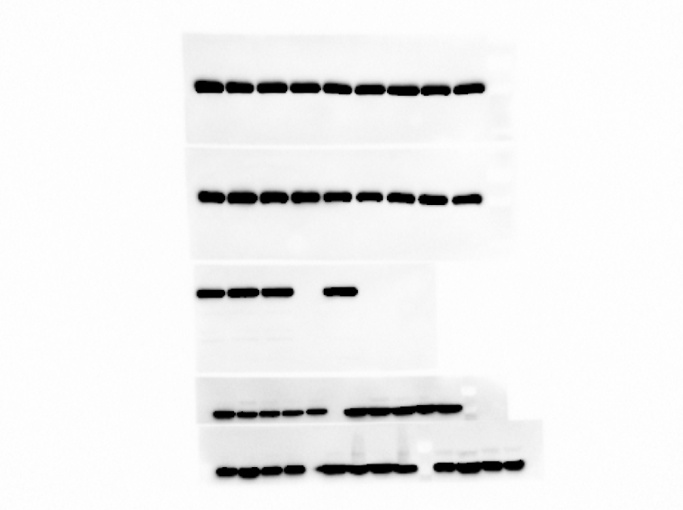


U87MH: P-AKT(S473) P-PRAS40(T246) GAPDH


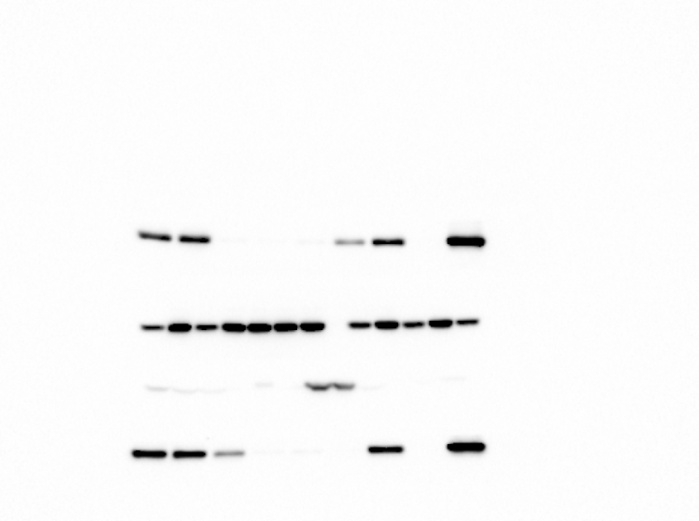

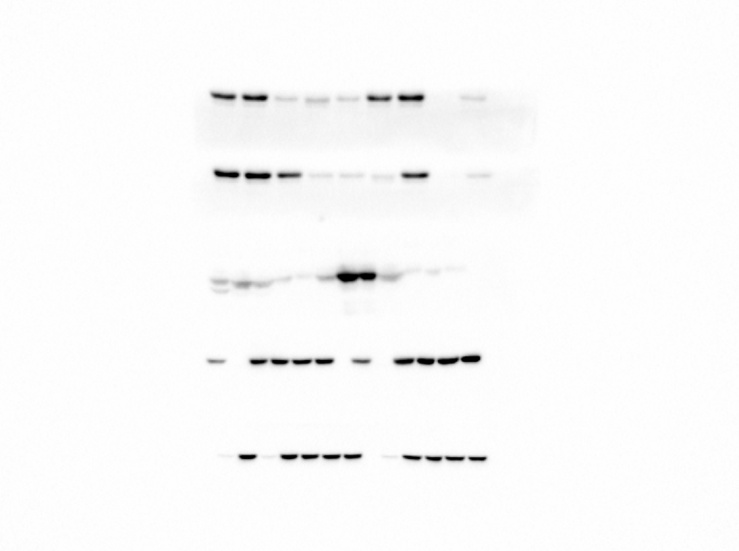

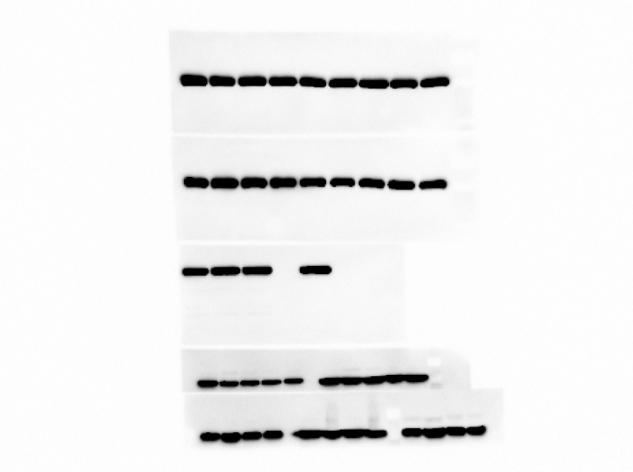


Fig5D:

LN-229-TRIP13: CHOP GAPDH


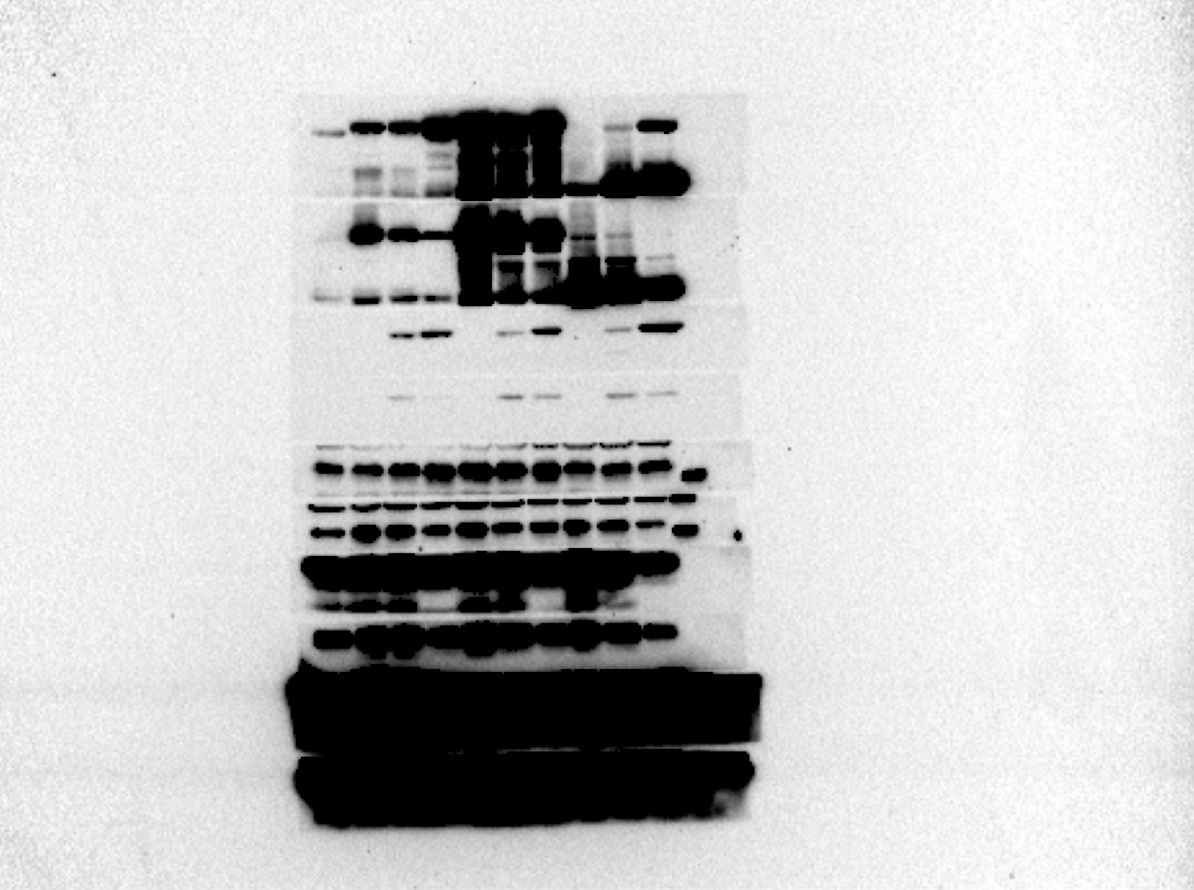

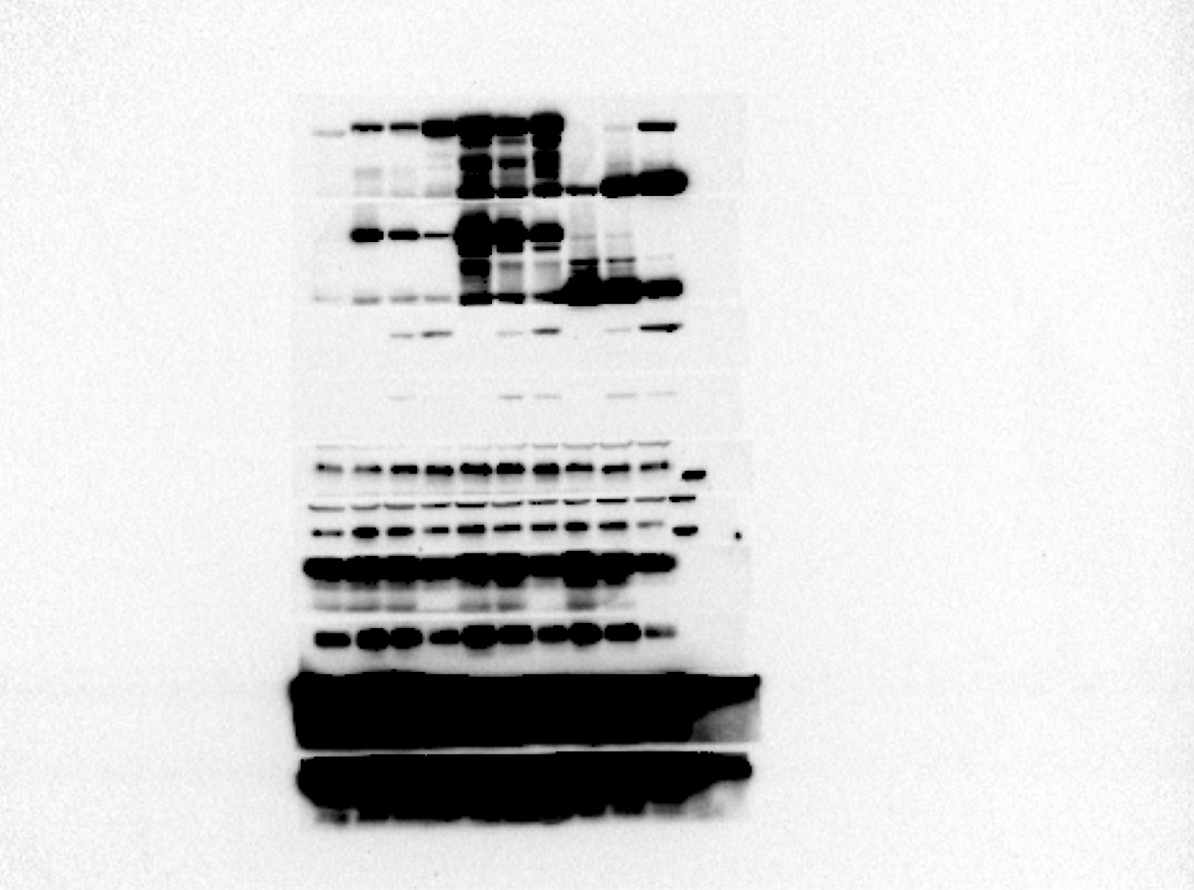


LN-18-TRIP13: CHOP GAPDH


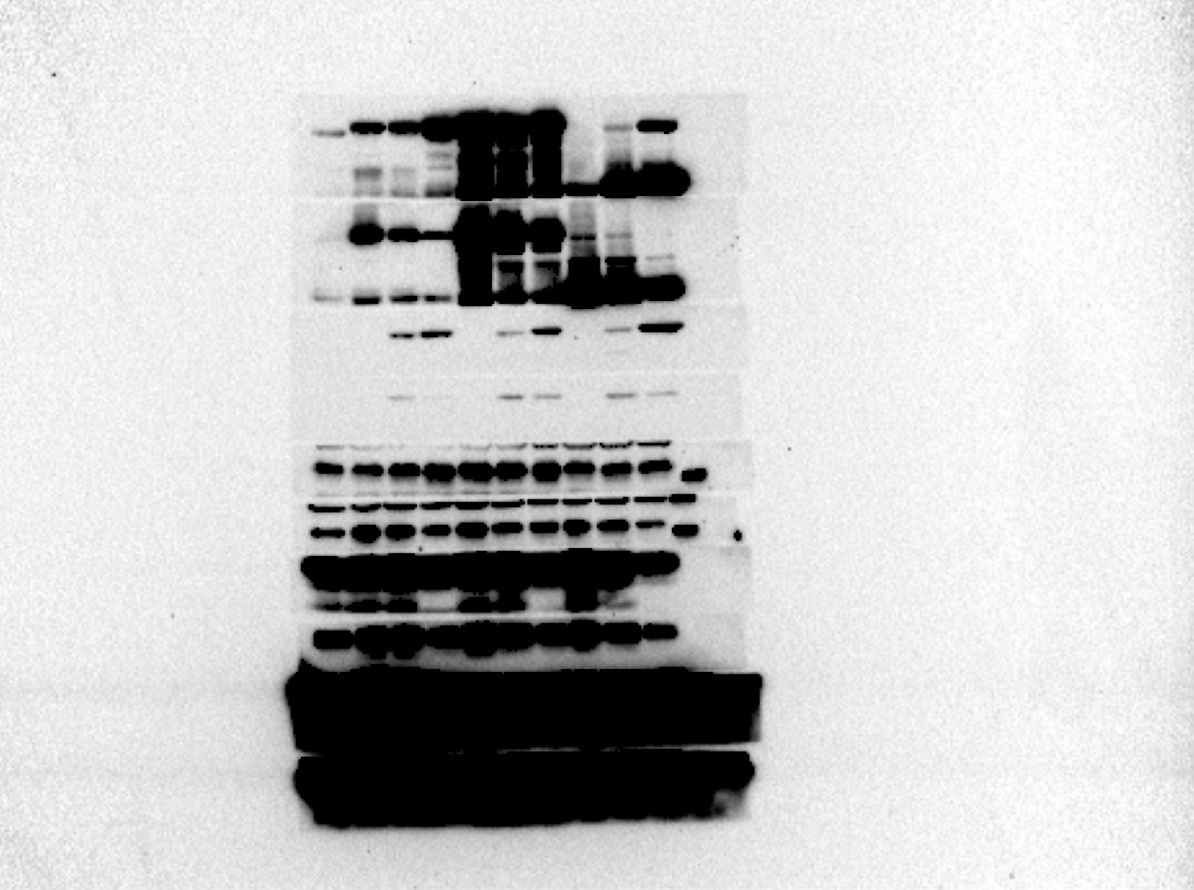

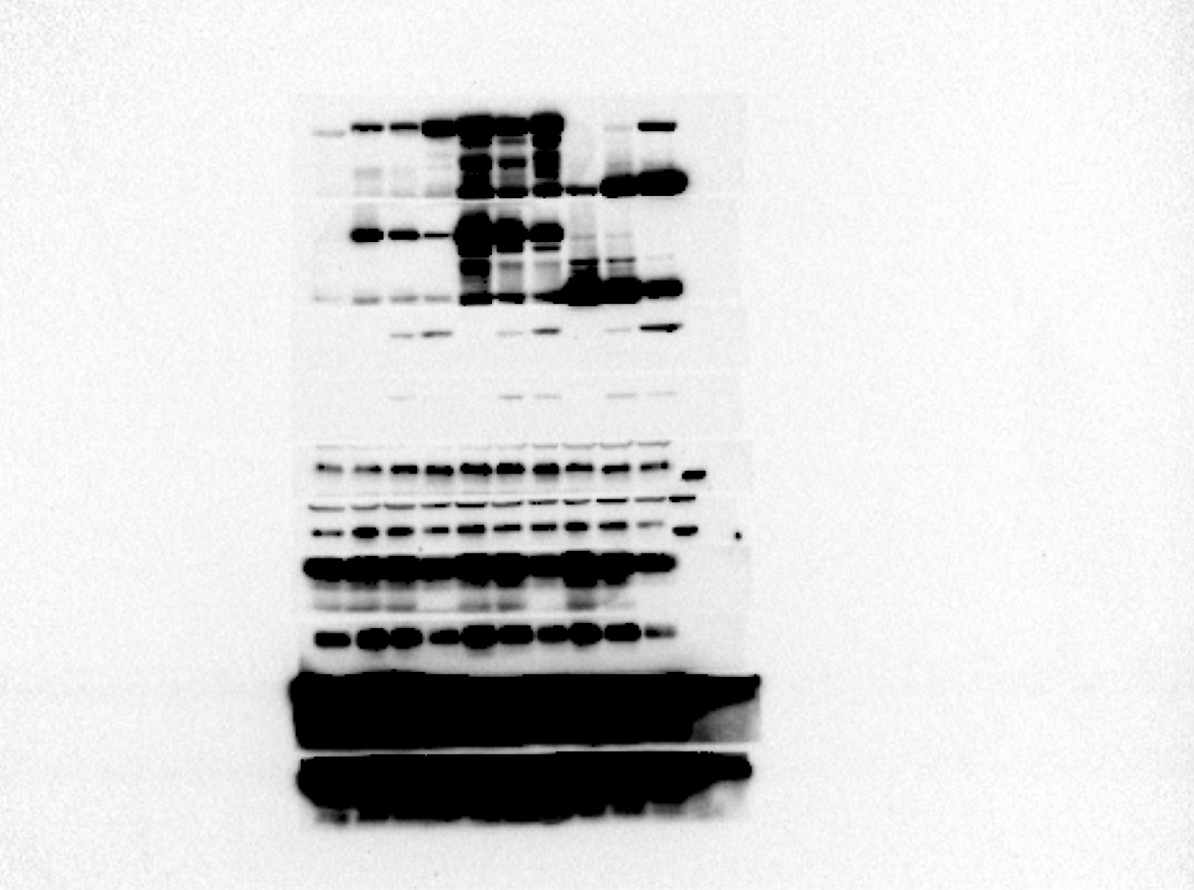


Supplementary1A:

U87MG:Caspase-3 PARP EGFR GAPDH


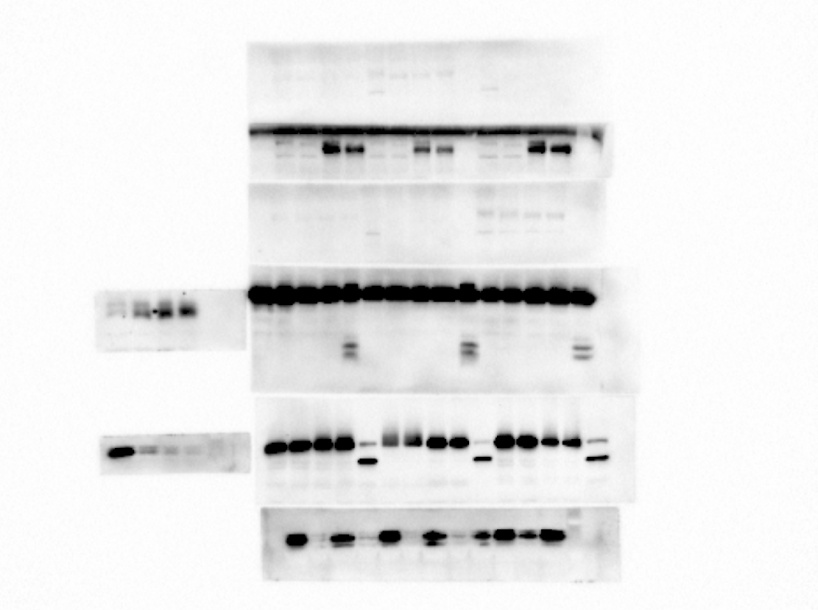

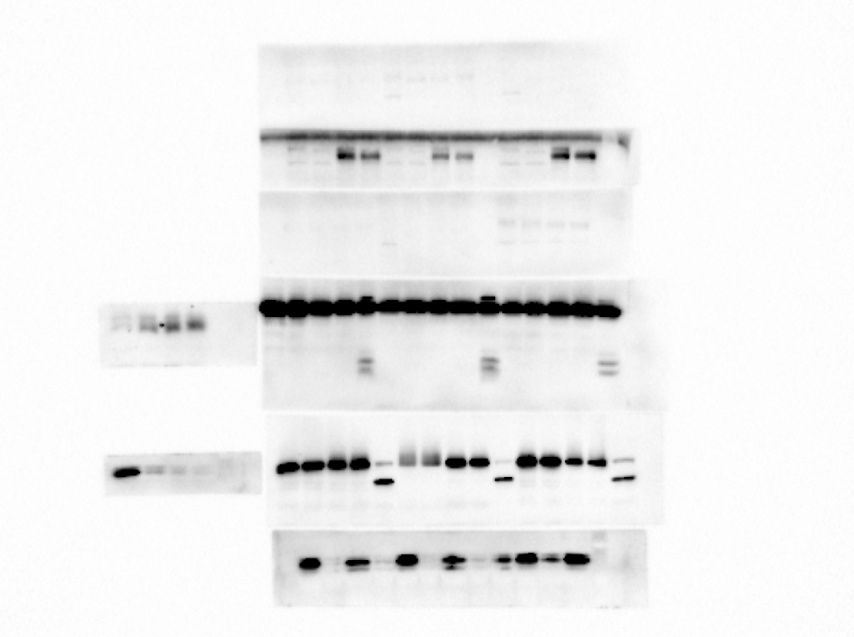

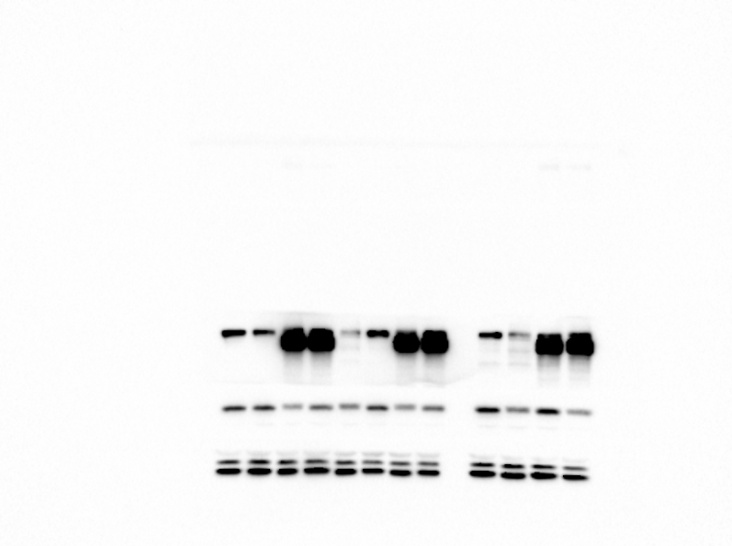

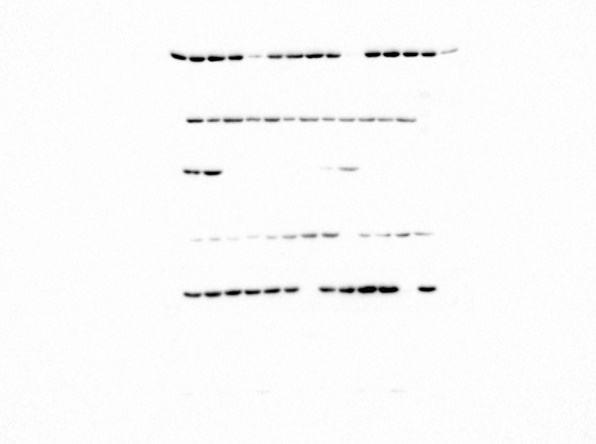


LN-229: Caspase-3 PARP EGFR GAPDH


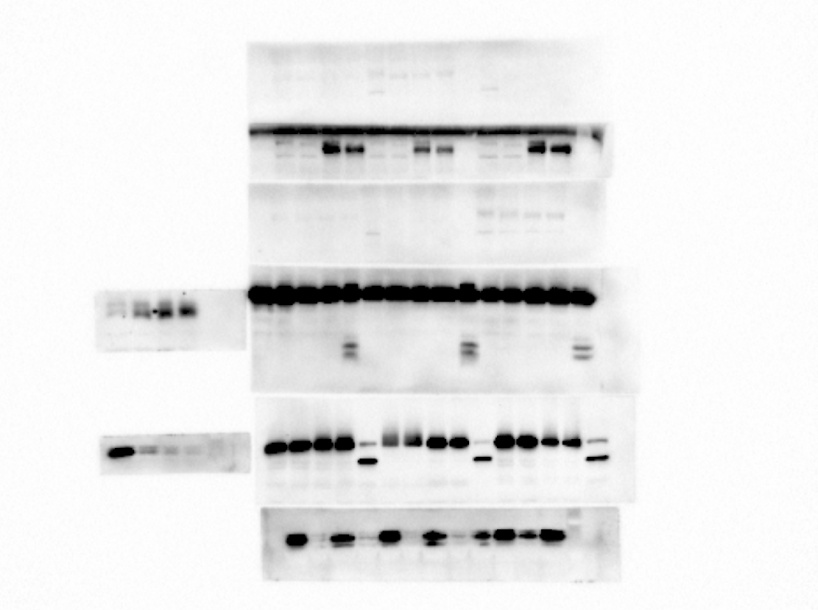


Supplementary1C:

LN-229:P62 LC3 GAPDH

U87MG: P62 LC3 GAPDH

Supplementary1I:

LN-229: CHOP GAPDH

U87MG: CHOP GAPDH
